# LOX and Its Methylation Impact Prognosis of Diseases and Correlate with TAM Infiltration in ESCA

**DOI:** 10.1155/2022/5111237

**Published:** 2022-08-31

**Authors:** Chuanqiang Wu, Bin Jiang, Zixiang Wu, Lingjun Dong, Keyi Sun, Ming Wu

**Affiliations:** ^1^Department of Thoracic Surgery, The Second Affiliated Hospital of Zhejiang University School of Medicine, 88 Jie-Fang Road, Hangzhou 310009, Zhejiang, China; ^2^Department of Thoracic Surgery, Shaoxing People's Hospital, 568-Zhongxing North Road, Shaoxing 312000, Zhejiang, China

## Abstract

**Background:**

ESCA is one of the digestive tract tumors with a high fatality. It is implicated in an intricate gene regulation process, but the pathogenesis remains ambiguous.

**Methods:**

The study used the packages of Limma from R software to analyze DEGs of ESCA in the GEO database and TCGA database. We employed the DAVID website for enrichment analysis, and the string database constructed the PPI network. Hub genes were identified from ESCA DEGs with Cytoscape MCODE. We evaluated the clinical relevance of LOX expression and its DNA methylation in the cBioPortal database and explored the roles of LOX in ESCA immunity, especially immune cell infiltration levels and immune checkpoint expression, by immunedeconv package of R software.

**Conclusions:**

The overexpression of LOX in ESCA is regulated by DNA hypomethylation; LOX overexpression or LOX hypomethylation can predict a worse prognosis in patients with ESCA. Besides, LOX may be involved in TIME regulation, promoting the infiltration levels and function of TAM. Hence, high LOX expression affected by DNA hypomethylation has an essential role in patients with ESCA, which may become an effective prognostic marker and therapeutic target.

## 1. Introduction

Esophageal cancer (ESCA) is the 7th most frequent malignant tumor and the 6th leading cause of cancer-related death, especially in Asia. There were approximately 570,000 new cases of ESCA in 2018, resulting in more than 500,000 cancer deaths worldwide [1]. The categories of the pathological subtypes of ESCA include esophageal squamous cell carcinoma (ESCC) and esophageal adenocarcinoma (EAC) [2]; the former accounts for almost 90% of all cases [3]. The 5-year overall survival rate for ESCA ranges from 15% to 25% [2], and the prognosis is largely dependent on early diagnosis of the disease [4]. The analysis of comprehensive mutations using high-throughput sequencing technology has conformed extensive genomic alterations in ESCA [5, 6], The sensitivity of the currently used markers SCC, CEA, and CYFRA 21-1 is only 20% to 50% [7], while other common variants, such as TP53, RB1, and CDKN2A [8], are still far from being widely used in clinical practice. Therefore, further exploration of molecular variants that influence esophageal cancer initiation and progression may unveil new insights for early diagnosis of the disease and the formulation of personalized treatment strategies.

Epigenetic modifications are heritable changes to regulate the cellular gene expression patterns, which are necessary for the appropriate development and conservation of various tissue functions. DNA methylation, the most well-characterized epigenetic mechanism that plays a pivotal role in governing gene expression and maintaining genome stability [9, 10], has been linked to cancer as early as 1983 [11], which mainly occurs in a cytosine-phosphate-guanine (CpG) dinucleotide context, with an amount of evidence demonstrating that DNA methylation inhibits transcription [9, 10]. There is reason to believe that DNA methylation has an influential role in the control of distinct gene expression and tumor development; still, the effects of DNA hypomethylation on tumorigenesis are poorly understood [12, 13].

Cancer immunotherapy has made great strides and reached a milestone that has completely transformed the treatment options for several primary cancers, including ESCA [14–16]. Although many researchers believe that immunotherapy is new hope for many cancers, a considerable number of patients have been found to have relapsed or acquired symptoms [17]. Given the moderate antitumor efficacy and relatively widespread drug resistance of immunotherapy, immunotherapy combined with other therapies aims to recruit more immune cells to enter the tumor or activate tumor-killing immune cells, which is considered an effective way to improve the therapeutic efficiency [18].

Over the past few decades, bioinformatic (differentially expressed genes) screening has assisted us in identifying hub genes and pathways involved in tumorigenesis and progression. This research aims to understand major altered molecular events of ESCA and find new targets for clinical application. Firstly, DEGs were screened between normal and tumor tissues, and a hub gene LOX was identified. Secondly, we discovered that LOX expression in ESCA is affected by methylation and related to clinicopathological features. Finally, we investigated the association between LOX expression and the ESCA tumor immune microenvironment (TIME). Furthermore, preliminary validation data in clinical samples validate the bioinformatic findings.

## 2. Materials and Methods

### 2.1. Data Sources

In the GEO database, we downloaded four ESCA gene datasets (GSE20347, GSE23400, GSE38129, and GSE67269), including tumor and corresponding adjacent normal tissue samples. GSE20347, GSE23400, GSE38129, and GSE67269 contain 17, 53, 30, and 73 cases, respectively. Besides, standardized gene expression data and clinical observation data for 162 patients were extracted from The Cancer Genome Atlas (TCGA, https://www.cancer.gov/about-nci/organization/ccg/research/structural-genomics/tcga). Normal tissue sample expression data from the GTEx V8 release version (https://gtexportal.org/home/datasets) were downloaded. For a complete description of donor gender, race, age, tobacco, and alcohol use, see the GTEx Official Notes.

### 2.2. Identification of DEGs and PPI Network Construction

Limma package in R studio software (Version 1.3.1073) was applied to analyze DEGs. Adjusted *p* values were analyzed to correct false positives across the three databases. DEG screening was defined with a threshold of “Adj *p* < 0.05 and Fold Change >2 or< −2.” PPI network was forecasted with a web-based STRING database and drawn in Cytoscape software (version 3.8.0, The Cytoscape Consortium, New York, NY). The MCODE clustering algorithm in Cytoscape was employed to find the most prominent dense module and hub gene.

### 2.3. KEGG and GO Enrichment Analyses of DEGs or LOX-Related Gene

The functional analysis of genes and biological pathways was conducted with the Database for Annotation, Visualization, and Integrated Discovery databases (DAVID; https://david.ncifcrf.gov/home.jsp).

### 2.4. Survival Analysis

The survival differences between the groups were compared with KM survival analysis and log-rank test. For Kaplan–Meier curves, *p* values and hazard ratio (HR) were generated by log-rank tests and univariate Cox proportional hazards regression. For differences in survival between the two groups, KM survival analysis and log-rank tests were performed.

### 2.5. MEXPRESS Analysis

We used MEXPRESS (https://mexpress.be/) databases to examine the ESCA TCGA DNA methylation data of different CpGs and the correlation of DNA methylation data with gene expression and several clinical factors.

### 2.6. Methylation Survival Analysis

We used the SurvivalMeth database (https://bio-bigdata.hrbmu.edu.cn/survivalmeth/) to study the function of DNA methylation-associated items and the relationship between ESCA patient's survival and the methylation level of LOX.

### 2.7. cBioPortal Analysis

The cBio Cancer Genomics Portal (cBioPortal; https://www.cbioportal.org/) was used to explore the cancer genomic methylation data, comparing the methylation differences between different groups of ESCA.

### 2.8. TIMER Analysis

Tumor Immune Estimation Resource (TIMER, https://cistrome.shinyapps.io/timer/) is a comprehensive website for automatically analyzing and visualizing the relationship between immune infiltration levels and various variables. We assessed the correlation of LOX expression with TAM infiltration levels, M1 type macrophage marker, M2 type macrophage marker, and related factors in ESCA by the TIMER algorithm. We also explored the predictive value of LOX and macrophage abundance in patients with ESCA using the TIMER database.

### 2.9. Definition of immune Subtypes

Original data of 162 ESCA samples were extracted from the TCGA database, 1959 immune-related gene expression profiles were assessed, and a consensus cluster was built to identify corresponding immune subtypes and gene modules. The maximum number of clusters was 6, and 80% of the total sample was drawn 100 times, cluster Alg = “hc,” innerLinkage = “ward. D2.” Clustering heatmaps were generated using the “pheatmap” R package (v1.0.12). The gene expression heatmap reserved genes with SD > 0.1. When the number of input genes exceeds 1000, the top 25% of genes are extracted after SD sorting.

### 2.10. Analysis between TMB, Immune Checkpoint, and LOX Expression Values

Correlations between quantitative variables were determined using Spearman analysis. Eight gene expression levels were extracted from the TCGA database as immune checkpoint scores, including CD274, CTLA4, HAVCR2, LAG3, PDCD1, PDCD1LG2, TIGIT, and SIGLEC15.

### 2.11. Immune Infiltration Estimations

Reliable estimates of immune infiltration were made using an R package: immunedeconv, including Cibersort, EPIC, MCP-counter, QUANTISEQ, and TIMER algorithm.

### 2.12. Sample Collection

All tissue samples were collected from surgical excision specimens in the Second Affiliated Hospital Zhejiang University School of Medicine (SAHZU); all tumors were histopathologically confirmed as ESCA. All specimens were collected according to guidelines approved by the institutional review board at the SAHZU.

### 2.13. Western Blot Analysis

For tissue protein extracts, 10 mg of frozen tumor and normal tissues were ground in a mortar on ice and lysed with RIPA lysis buffer. Protein concentration was detected by the BCA method. Proteins were isolated on 10% SDS-PAGE and then blotted onto the PVDF membrane. After being blocked in 5% skim milk, the membrane was incubated with rabbit anti-human LOX (HUABIO, cat.no. ET1706-31) and mouse anti-human GAPDH antibodies (Proteintech, cat. no. 60004-1-Ig) at 1 : 1000, 12 hours at 4 degree Celsius. After washing, the membrane was incubated with corresponding secondary antibodies (1 : 10,000; proteintech). The ECL system was used for the immunoreactive bands' defection. Band gray analysis was measured by ImageJ software.

### 2.14. Methylation-Specific Polymerase Chain Reaction (MSP)

The method was conducted as described previously [19]. MSP primers were generated from genomic sequences surrounding the transcription start site (TSS) and synthesized to detect unmethylated (U) and methylated (M) alleles. The thermal cycling parameters were as follows: 95 degree Celsius 10 min; (95% 45 s, 58 degree Celsius 30 seconds, and 72 degree Celsius 40 seconds), 35 cycles; 72 degree Celsius 8 min. About 10 *μ*l of PCR products was running in 3% agarose gels and visualized using the ethidium bromide staining method.

### 2.15. Statistical Analysis

The above data were analyzed by R and R Studio software (Version 1.3.1073). The groups with low and high LOX expression were determined according to the median LOX mRNA expression in different datasets. According to the grouping strategy defined by websites, LOX hypomethylation and hypermethylation groups were established. The Cox proportional hazards regression model was used to calculate the hazard ratio. The relationship between LOX expression or DNA methylation and a series of taxonomic factors was calculated using the *t*-test or the Mann–Whitney test. *p* < 0.05 was set as the statistically significant threshold.

## 3. Results

### 3.1. Characterization of DEGs and Associated Signaling Pathways in ESCA

**Figure 1:**
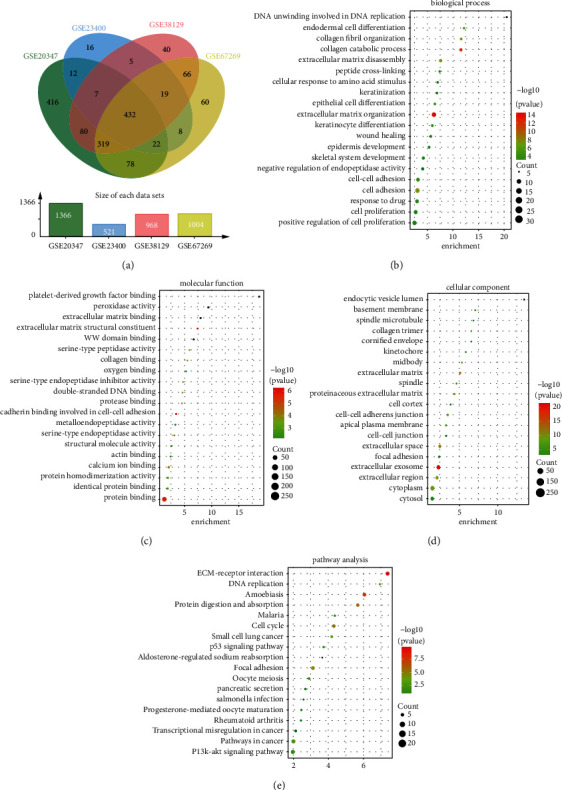
Differential expression genes and related biological pathways in the GEO-ESCA cohort. (a) DEGs with fold changes >2 and *p* values <0.05 were selected in four mRNA expression profiling of GSE20347, GSE23400, GSE38129, and GSE67269. The 4 datasets show that 432 genes overlap. Kyoto Encyclopedia of Genes and Genomes (KEGG) biological process (b), molecular function (c), cellular component (d), and pathway enrichment analysis (e) of DEGs from the GEO-ESCA cohort.

To clarify the molecular mechanism and find essential factors affecting the occurrence and development of ESCA, four datasets (GSE20347, GSE23400, GSE38129, and GSE67269) were downloaded from the GEO database; after normalizing the expression data, we identified differential genes in these datasets (1,366 in GSE20347, 521 in GSE23400, 968 in GSE38129, and 1,004 in GSE67269) (Supplementary Table 1). The Venn diagram shows the overlap of 432 DEGs between these four datasets (Figure 1(a)), including 217 downregulated genes and 215 upregulated genes (Supplementary Table 2). Then, we performed function and pathway enrichment analysis to decipher the biological process of DEGs. GO analysis disclosed the involved biological process (BP) of DEGs, mainly enriched in an extracellular matrix organization, cell adhesion, skeletal system development, and cell proliferation (Figure 1(b)). Molecular function (MF) changes are primarily concentrated in protein binding, cell adhesion, protease activity, and extracellular matrix structural constituent (Figure 1(c)). Cellular composition (CC) changes are mainly accumulated in extracellular exosomes, the extracellular space, and the extracellular matrix (Figure 1(d)). The analysis of the KEGG signaling pathway revealed that DEGs were involved primarily in ECM-receptor interaction, pathways in cancer, and p53 signaling (Figure 1(e)). We analyzed the ESCA data and explored the differential genes in the TCGA and GTEX cohorts to verify the results (Figures S1A and S1B) and performed KEGG and GO enrichment analyses. The p53 signaling pathway, transcriptional misregulation in cancer, cell-cell adhesion, and T-cell activation regulation were enriched more significantly (Figures S1C and S1D). In contrast, the muscle system process, focal adhesion, and extracellular matrix organization were enhanced more significantly (Figures S1E and S1F). In conclusion, the results obtained from the GEO and TCGA databases were consistent. The crucial roles for p53 signaling, disruption of tumor transcription, intercellular adhesion, and extracellular matrix organization in the ESCA tumorigenesis and progression have been described [6,20–25]. Besides, there were also studies on the relationship between the muscle system and ESCA [26, 27]. In recent years, the idea of the ESCA immune microenvironment that regulates antitumor immunity has also attracted the increasing attention of researchers [15, 16, 28]. However, it remains unclear how the immune microenvironment modulates the antitumor immune function of ESCA. Taken together, all these studies reflect that our results are worthwhile.

### 3.2. Prognostic Value and Clinicopathological Features of LOX Expression according to the TCGA Database

**Figure 2:**
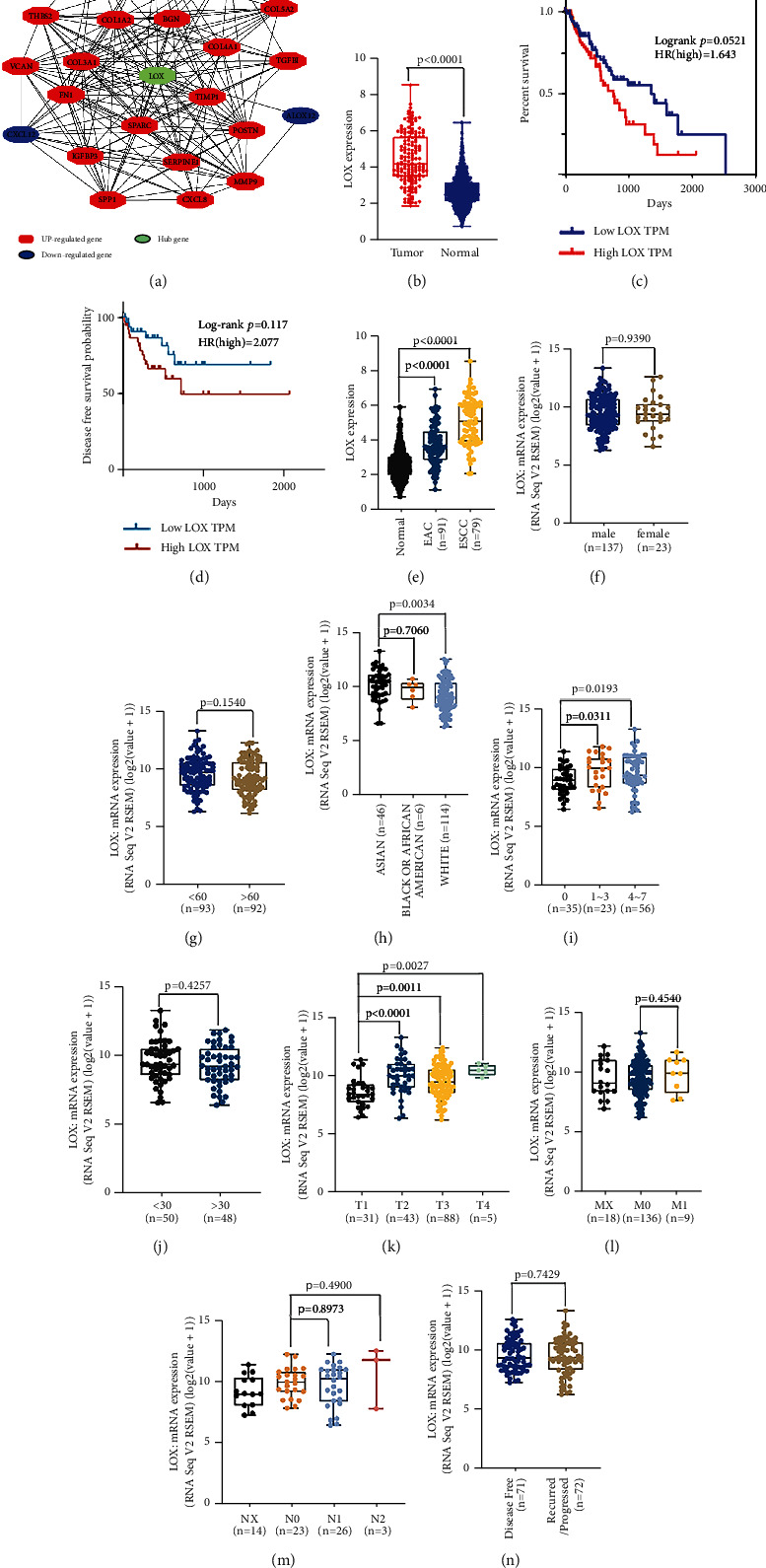
Correlation between LOX expression and clinicopathological features in ESCA patients. (a) The LOX PPI network was built with Cytoscape. (b) Higher expression of LOX mRNA in ESCA tissues in the TCGA dataset. (c) Kaplan–Meier survival curve and Cox regression model of different LOX expression statuses in ESCA patients. (d) DFS of low and high LOX expression in ESCA patients. The mRNA expression of LOX stratified by histology type (e); gender (f); age (g); race (h); alcohol consumption frequencies (i); person cigarette smoking history pack-year (j); tumor stage (k); lymph node stage (l); metastasis stage (m); disease-free status (n). Each point represents a patient.

We submitted DEG symbols to the web-based STRING database for PPI interaction network analysis and searched hub genes with Cytoscape MCODE. A total of four hub genes (LOX, IFI44, IL18, and SLURP1) were identified (Figures 2(a) and S2A). TCGA database analysis revealed that LOX and IFI44 mRNA expressions were higher in ESCA tumor tissues, while IL18 and SLURP1 mRNA expressions were higher in normal tissues (Figure 2(b), Figures S2B–S2D). Survival analysis confirmed that LOX expression levels were inversely correlated with long-term survival and disease-free survival (DFS) of ESCA (Figures 2(c) and 2(d)); however, there was no significant correlation between the gene expression levels of the other three genes and survival (Figures S2E–S2G). Next, we compared the differential expression of LOX mRNA among groups from the TCGA database divided by histology type, gender, age, race, alcohol consumption frequencies, person cigarette smoking history pack-year, T stage, N stage, M stage, and DFS (Supplementary Table 4). Results showed a tie-in between LOX expression and histology type (*p* < 0.0001, *p* < 0.0001) (Figure 2(e)), race (*p*=0.0034, *p*=0.7060) (Figure 2(h)), alcohol consumption frequencies (*p*=0.0311, *p*=0.0193) (Figure 2(i)), and T stage (*p* < 0.0001, *p*=0.0011, *p*=0.0027) (Figure 2(k)), indicating the close relationship of LOX expression with a set of clinical parameters. Whereas the expression of LOX mRNA is not related to gender (*p*=0.9085) (Figure 2(f)), age (*p*=0.1540) (Figure 2(g)), patients smoking habit (*p*=0.4527) (Figure 2(j)), lymph node stage (*p*=0.8973, *p*=4900) (Figure 2(m)), metastasis stage (*p*=0.4540) (Figure 2(l)), and disease-free status (Figure 2(n)). Thus, LOX may be a crucial factor in the formation and development of ESCA.

### 3.3. Analysis of LOX-Related Signaling Pathways in ESCA

To better understand the biological functions of LOX, we performed GO and KEGG enrichment analysis to explore the potential biological processes of LOX-related genes in ESCA. We found that LOX-related genes mainly focus on ECM-receptor interaction, cell adhesion, focal adhesion, cell proliferation, differentiation, transcription regulation, and extracellular matrix organization (Figures S3A and S3B). Among them, upregulated genes are mainly enriched in pathways related to glucose and lipid metabolism, cell adhesion, and immune response (Figures S3C and S3D), and downregulated genes enhanced primarily on signaling pathways for CGMP-PKG, TGF-*β*, WNT, PI3K/AKT, extracellular matrix organization, and cell-cell adhesion (Figures S3E and S3F). The results showed that the enrichment results of LOX-related genes are similar to the enrichment results of ESCA DEGs in cell-cell adhesion, extracellular matrix organization, ECM-receptor interaction, transcription regulation, and immune response. In a word, LOX implicated multiple biological processes and played a crucial role in ESCA.

### 3.4. DNA Methylation Level of LOX Correlated with LOX mRNA Expression

**Figure 3:**
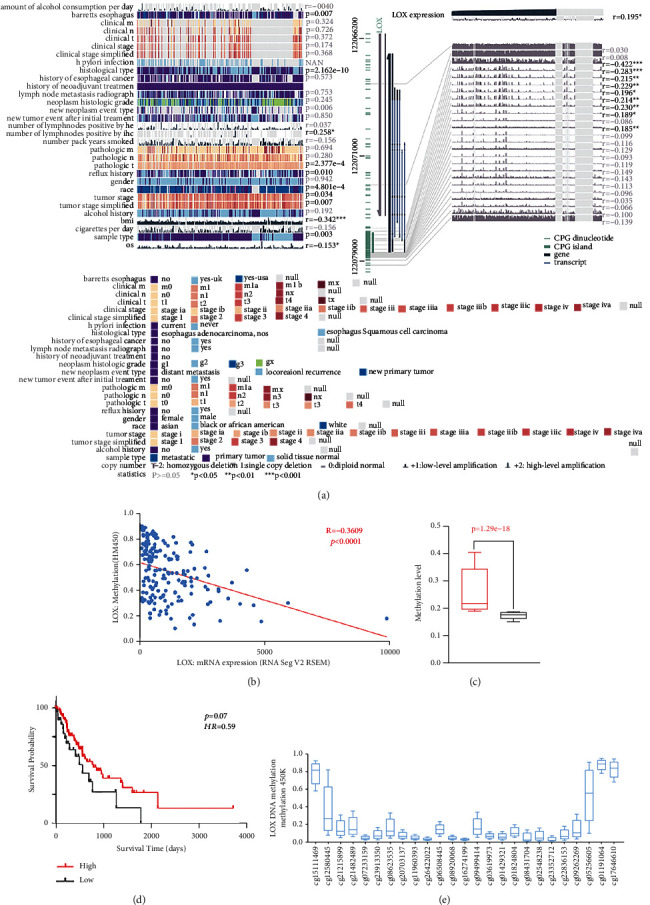
Clinicopathological features and methylation of LOX in ESCA tumor tissues and adjacent normal tissues. (a) The clinicopathological features and methylation of LOX. (b) The Pearson correlation coefficient of LOX expression with LOX DNA methylation level. Differences in methylation level (c) and OS (d) between hypomethylation group and hypermethylation group. (e) The distribution of 25 LOX DNA methylation sites.

We employed the MEXPRESS database further to verify the correlation between clinicopathological parameters and LOX expression, results showing that LOX expression is related to Barrett's esophagus, histological type, the number of lymph nodes positive, pathological T stage, reflux history, race, tumor stage, BMI, sample type, and OS (Figure 3(a)), which is consistent with the analysis results in Figure 2. Besides, we could observe a negative correlation between LOX expression and LOX DNA methylation level (*R* = −0.3609,*p* < 0.001) (Figures 3(a) and 3(b)). Then, we interpreted the relationship between LOX DNA methylation level and survival of patients with ESCA, indicating that LOX hypomethylation was related to worse overall survival (Figures 3(c) and 3(d)). The distribution of Tween-5 LOX global DNA hypomethylation sites is exhibited in Figure 3(e). As shown in Figure 3(a), among 25 methylation sites in the LOX gene, 23 of them were negatively correlated with LOX expression; 9 sites (cg05256605, cg09262269, cg22836153, cg23352712, cg02548238, cg08431704, cg01824804, cg01429321, and cg09499414) showed a significant correlation in ESCA. To understand the prognostic effect of LOX methylation at these sites in patients with ESCA, we applied KM survival analysis to study the relationship between these methylation sites and OS, suggesting that cg05256605 and cg09262269 hypermethylation are related to a better prognosis, while cg23352712 hypermethylation is a worse prognostic factor for patients with ESCA (Figure S4).

### 3.5. Clinical Significance of LOX Methylation Level According to the TCGA Database

Then, we explored LOX methylation levels with clinicopathological features and prognosis. Differences in LOX methylation levels in each group were studied according to histological type, gender, age, race, alcohol consumption frequencies, person cigarette smoking history pack-year, tumor stage, lymph node stage, metastasis stage, and disease-free status (Supplementary Table 4). As clearly illustrated in Figure S5, there were significant statistical differences between groups in terms of histology type (*p* < 0.0001) (Figure S5A), age (*p*=0.0105) (Figure S5C), race (*p*=0.7060, *p*=0.034) (Figure S5D), alcohol consumption frequency (*p*=0.0062, *p*=0.0023) (Figure S5E), and tumor stage (Figure S5G) (*p*=0.0005, *p*=0.0009, *p*=0.1610), demonstrating that LOX methylation level is related to a series of essential clinical features. Interestingly, clinicopathological parameters related to LOX mRNA expression levels are also meaningful in the methylation group, showing a negative correlation to a certain extent, indicating the very close relationship between the LOX DNA methylation and expression.

### 3.6. Experimental Data Confirmed the Bioinformatic Analysis Results

**Figure 4:**
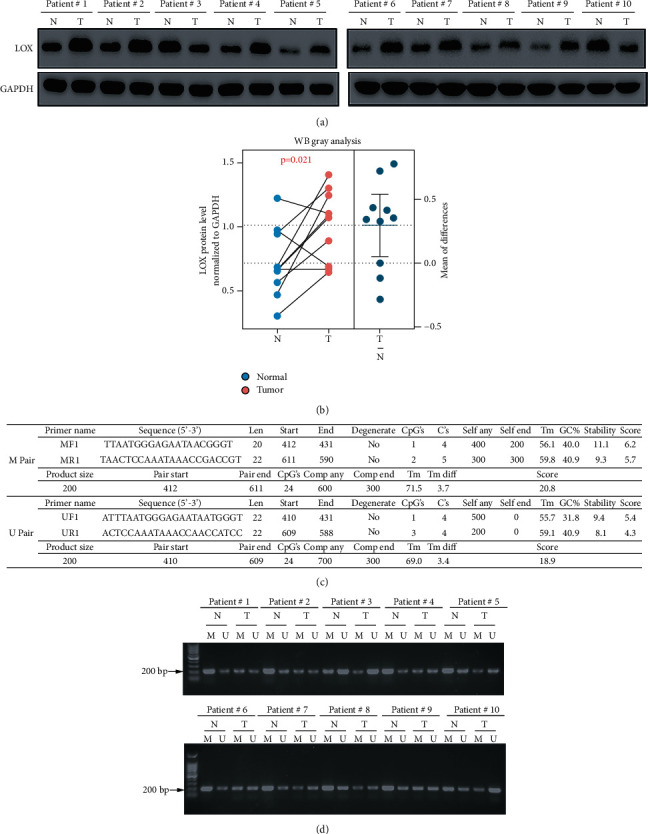
The ESCA tissues' validation data of the bioinformatic analysis results. (a) Western blot analysis of protein level of LOX with GAPDH as an internal reference. Tumor tissues (T) and adjacent normal tissues (N). (b) The protein expression level of LOX was measured by ImageJ software (c) The methylation-specific primer and unmethylation-specific primer were designed on Methypimer database. (d) Methylation-specific polymerase chain reaction (MSP) results in patients with ESCA.

To verify the above results, we detected the protein expression level of LOX in human ESCA specimens. The result suggested that the expression of LOX is profoundly higher in most (8/10) tumors than in normal tissues (Figures 4(a) and 4(b)). Next, we determined the methylation level of LOX promoters in these samples by using MSP. The methylation-specific primer (M) and unmethylation-specific primer (U) are shown in Figure 4(c). Methylated bands were faint in tumor tissues and more pronounced in normal tissues, thus confirming the low methylation level of LOX in tumors compared to normal tissues (Figure 4(d)).

### 3.7. Identification and Evaluation of the Association of Immune Status with LOX Expression

**Figure 5:**
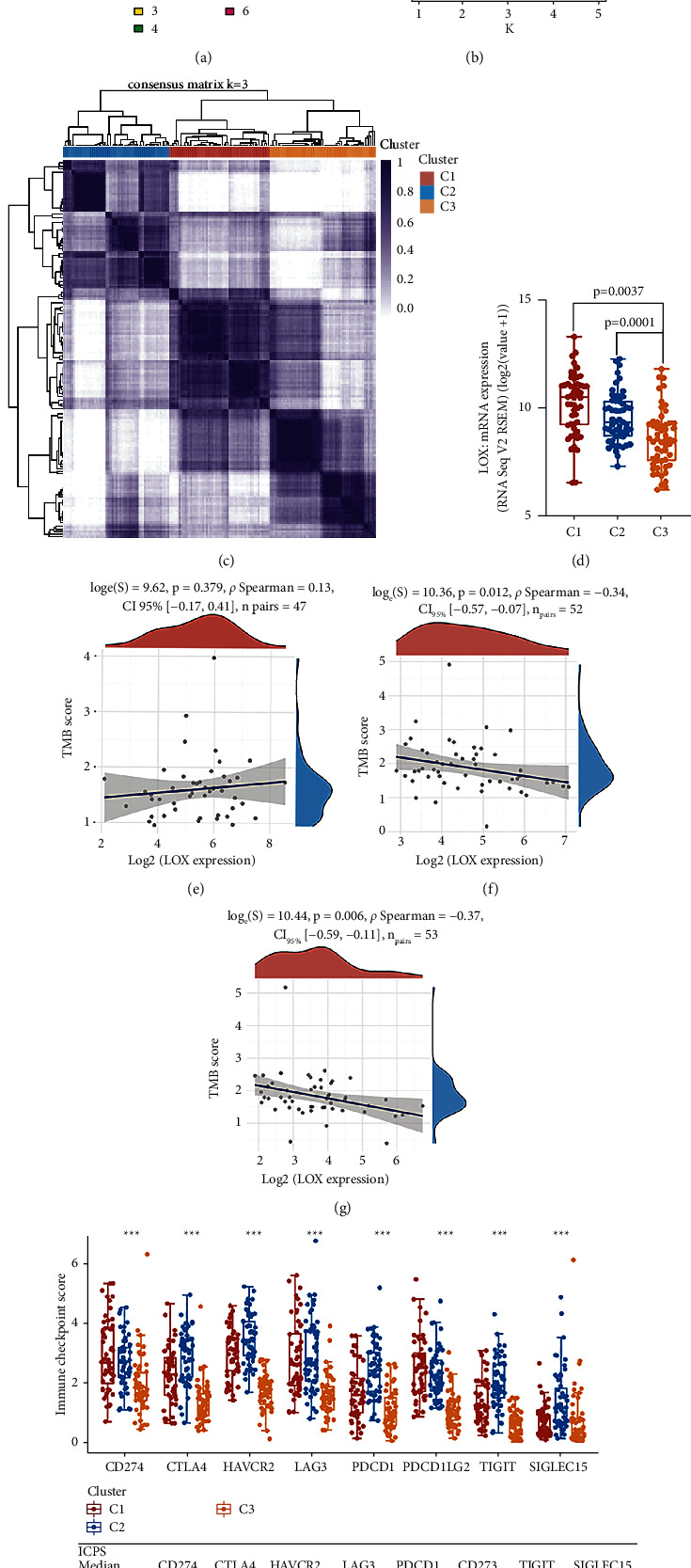
Correlation between LOX mRNA expression and immune subtypes, TMB, ICP of ESCA patients. (a) Consensus CDF. Delta area curve (b) of consensus clustering. (c) Cluster consensus (*k* = 3) heatmap of 1959 immune-related genes in 162 samples. (d) LOX mRNA expression in each cluster. Relationship between LOX expression and TMB among three clusters: C1 (e); C2 (f); and C3 (g). (h) The relationship between LOX expression and eight ICP expression (CD274, CTLA4, HAVCR2, LAG3, PDCD1, CD273, TIGIT, and SIGLEC15) among three clusters.

**Figure 6:**
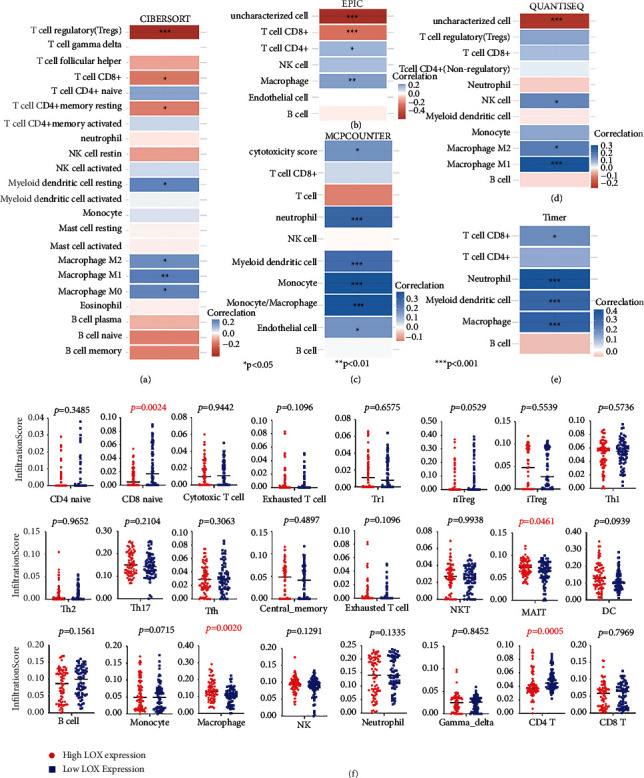
Immune cell infiltration estimation for ESCA tumors. Immune cell infiltration for ESCA tumors from TCGA database by CIBERSORT (a); EPIC (b); MCPCOUNTER (c); QUANTISQ (d); and TIMER (e). (f) Immune cell abundance identifier was applied to calculate the abundance of 24 immune cell types among different LOX expression groups from GEO-ESCA database.

As a new star in cancer treatment, in recent years, immunotherapy has shown encouraging effects in the treatment of various malignancies [29, 30]. Several clinical studies on immunotherapy for patients with ESCA are ongoing, and preliminary research results suggest that immunotherapy has considerable potential in treating ESCA [31]. In the enrichment analysis of KEGG and GO, our results also showed that immune response is highly significant in ESCA. Nowadays, with advances in technology, immune subsets are being described and classified with unprecedented precision, and their impact on disease development is being understood [30]. We, therefore, created consensus clusters based on 1959 immune-related gene expression profiles in 162 ESCA samples from the TCGA database. When *K* = 3, immune-related genes appear to be well clustered according to their cumulative distribution of functional and functional delta regions (Figures 5(a) and 5(b)), and thus obtained 3 clusters termed as C1∼C3 (Figure 5(c)). We then analyzed LOX expression levels in distinct clusters, C1, C2, and C3, and found significant differences in LOX expression among different groups (*p*=0.0037, *p*=0.0001) (Figure 5(d)), suggesting a critical role for LOX in shaping the TIME. An index called tumor mutational burden (TMB) measures the total number of tumor mutations. Highly mutated tumors produce more neoantigens, making them more immunogenic, and therefore more responsive to immunotherapy [32]. Therefore, the same analysis was performed for the three immune subtypes in each patient using the TCGA mutation datasets processed by muta2. A positive relationship exists between LOX expression and TMB in cluster C1; however, there is a negative association in clusters C2 and C3 (Figures 5(e)–5(g)). We also compared eight immune checkpoint (ICP) expression (CD274, CTLA4, HAVCR2, LAG3, PDCD1, CD273, TIGIT, and SIGLEC15) status between 3 clusters. Kruskal–Wallis test confirmed that all ICPs in the three clusters were significantly different (Figure 5(h)). Except for the ICPs, the type, number, and function of immune cells are also linked to the effect of immunotherapy [16, 33]. As shown in Figure 6, we evaluated the immune cell infiltration level under different LOX expression states in the TCGA cohorts with the immunedeconv package, including five algorithms: Cibersort, EPIC, MCP-counter, QUANTISEQ, and TIMER. Wilcox test demonstrated that T-cell regulatory (Tregs), CD8+ T cell, CD4+ T cell, CD4+ memory resting T cell, myeloid dendritic cell resting, NK cell, M0 type macrophage, M1 type macrophage (M1), M2 type macrophage (M2), neutrophil, monocyte, endothelial cell, and myeloid dendritic cell were significantly different in at least one algorithm (Figures 6(a)–6(e)). Then, we analyzed immune cell infiltration levels in 4 ESCA GEO cohorts, showing that lower LOX expression levels were relevant to more infiltration of CD8 naïve cells, B cells, and CD4 T cells. In contrast, a higher LOX expression level was related to more infiltration of mucosal-associated invariant T cells (MAIT cells) and macrophages (Figure 6(f)). Our results demonstrated that LOX could potentially impact the ESCA immune cell infiltration.

### 3.8. LOX Affects the Infiltration and Function of TAM

**Figure 7:**
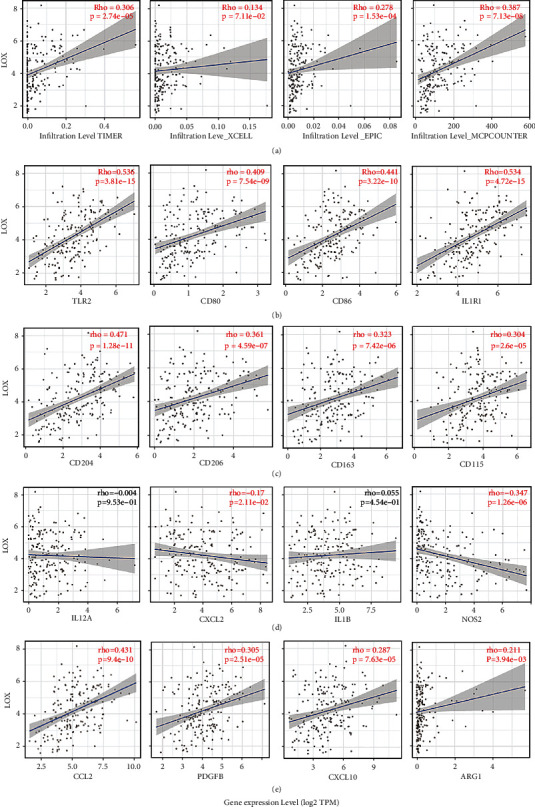
LOX mRNA expression level correlates with TAM infiltration in HCC. (a) The expression of LOX and the infiltration levels of macrophage are estimated by TIMER, XCELL, EPIC, and MCP-counter. LOX expression was correlated with biomarker expression of M1 type macrophage: TLR2, CD80, CD86, and ILR1 (b). M2 type macrophage: CD206, CD204, CD163, and CD115 (c). Soluble factors secreted by M1 : CCL2, PDGFB, CXCL10, and ARG1 (d). Soluble factors secreted by M2 : CCL2, PDGFB, CXCL10, and ARG (e).

The infiltration of immune cells into the TME is complex and plays different roles at different stages of cancer progression. Macrophages are more complicated cells in TME and have a more extensive effect on tumor progression [29, 34], promoting proliferation, invasion, and metastasis and causing cancer cells to develop immunotherapy tolerance called TAM [35, 36]. In Figure 6, we observed significant differences in macrophage infiltration levels in the GEO database and TCGA database under different LOX expression conditions. Then four algorithms, TIMER, XCell, EPIC, and MCP-counter, were employed to determine the relationship between LOX expression level and macrophage infiltration levels in the TCGA cohort. The result showed that higher LOX expression facilitated macrophage infiltration (Figure 7(a)), following the results of the GEO database. The macrophage can be activated or polarized differently in distinct TME to form subgroups with specific molecular and functional characteristics, mainly including M1, polymerization induced by IFN-*γ*, and M2, polymerization induced by IL4, IL10, or IL13 [34]. Our study found that the M1 polarization inducing factor, IFN-*γ*, and M2 polarization inducing factors, IL13 and IL10, were positively correlated with LOX expression (Figure S7B). M1 can kill tumor cells and resist pathogen invasion; M2 plays a vital role in promoting tumor growth, invasion, metastasis, and establishing an inhibitory immune microenvironment [35]. Polarization biomarkers for M1 include TLR2, CD86, CD80, and IL1R1 [36], and polarization biomarkers for M2 include CD163, CD204, CD206, and CD115 [37]. We analyzed the relationship between LOX expression and M1 and M2 markers in ESCA using the TIMER database. As exhibited in Figure 7, LOX expression is markedly correlated with M1 markers—TLR2, CD80, CD86, and IL1R1 (Figure 7(b))—and M2 markers—CD204, CD206, CD163, and CD115 (Figure 7(c)). Activated TAMs have been reported to significantly affect tumors through the direct production of soluble factors [35, 37]; therefore, we continue to study the relationship of LOX expression with soluble factors secreted by M1 and M2. The results of the TIMER database proved that the expression of LOX was positively correlated with soluble factors secreted by M2, such as CCL2, PDGFB, CXCL10, and ARG1 (7(e)), TGFB1, EGF, CCL22, MMP2, MMP9, MMP14, TNF, IL6 (Figure S6A). Secreted by M2. On the contrary, LOX expression was not correlated or negatively correlated with soluble cytokines, IL12A, CXCL2, IL1B, and NOS2, secreted by M1 (Figure 7(d)). Therefore, LOX may inhibit the factor secretion of M1 macrophage and promote the factor secretion of M2 macrophage.

### 3.9. LOX Expression Influences Prognosis of ESCA Patients through Immune Infiltration of TAMs

Since the LOX expression is connected to TAM's infiltration levels and related to the OS of ESCA, we speculated that the expression of LOX might influence the ESCA patients' prognosis by affecting the degree of TAM infiltration. Therefore, we further employed the survival curve analysis to verify our hypothesis with the XCEL and EPIC methods. The results indicated that lower macrophage infiltration was associated with a better prognosis of ESCA patients (Figures S7A, S7C, and S7E). Finally, we combined LOX expression levels and macrophage or M2-type macrophage infiltration levels for survival analysis. The analysis of the EPIC and XCELL methods showed that lower LOX expression and lower macrophage infiltration or lower M2 infiltration were more favorable for the prognosis of ESCA patients (Figures S7B, S7D, and S7F). These results suggested that LOX expression may influence ESCA progression through TAM immune infiltration.

## 4. Discussion

This study investigated four GEO-ESCA datasets and the TCGA-ESCA dataset to obtain DEGs between ESCA tumor tissue and corresponding adjacent normal esophageal tissue. Four hub genes were notified of which only the expression of LOX correlates with overall survival. Based on the TCGA database, we analyzed the relationship between LOX mRNA expression and methylation levels, immune infiltration, and clinical prognostic implications. Studied by bioinformatics and experiments, we found for the first time that LOX was highly expressed in ESCA tissues and there was a strong negative correlation between LOX mRNA expression and LOX methylation. Furthermore, we found that both LOX expression and LOX methylation were strongly associated with several critical clinical traits. Cox regression models identified the detrimental effects of LOX overexpression and hypomethylation on the prognosis of ESCA patients. Notably, we examined the association between LOX expression and ESCA immunity. Our analysis showed that the immune subtype, TMB, ICP expression, immune infiltration level, and TAM function were significantly related to LOX expression in ESCA. Our study of the potential biological roles and prognostic implications of LOX in ESCA sheds new light on elucidating molecular mechanisms and may offer new therapeutic options for ESCA.

In 1968, Pinnell and Martin discovered LOX [38], whose typical role is to catalyze the first step of covalent cross-linking of two significant extracellular matrix (ECM) proteins—collagen and elastin [39]. Growing evidence indicated that LOX promotes tumor formation and progression, such as glioblastoma multiforme, hepatocellular carcinoma, etc. [40–43]; in these studies, the overexpression of LOX is associated with tumor malignancy and poor prognosis. The expression of LOX is a valuable survival predictor for ESCC patients [44–46]. Our analysis results also showed that LOX expression is related to several clinical parameters, including histology, T stage, etc. Notably, it seems the T3 or T4 stage samples have lower LOX expression than those in the T2 stage. We considered that the abnormal LOX expression level in different T stages of ESCA might be related to the function of LOX in remodeling the structure of ECM. According to the T stage of esophageal cancer, stage T1 refers to tumor cell invasion into the lamina propria, muscularis mucosae, or submucosa; stage T2 refers to tumor cell invasion into muscularis propria; stage T3 refers to tumor cell invasion into adventitia; and stage T4 refers to tumor cells invading adjacent structures. The classically described function of LOX is that an enzyme catalyzes the cross-linking of collagen or elastin in the ECM and thereby regulates the tensile strength of the tissue. Connective tissues, which include elastic fibers and collagen, are distributed widely throughout the mucosa and submucosa of the esophagus. During the T1 stages, the expression level of the LOX gene increased gradually to remodel ECM, reaching a peak at the T2 stages. It may be progressively decreased after passing through the lamina propria and submucosa due to limited connective tissues in muscularis propria. Thus, when reaching the T3 stages, LOX expression is relatively high but lower than in the T2 stage. However, the oncogenic role of LOX in ESCC needs to be further verified. It was the first time finding the central function of LOX in ESCA development through the GEO database. In the TCGA database, LOX underexpression is highly relevant to better OS and DFS in ESCA patients. This conclusion was further supported by a Cox regression analysis, which demonstrated that LOX was a risk factor affecting OS and DFS in ESCA patients. In short, our research emphasizes that LOX is a hopeful biological marker for predicting prognosis in ESCA patients.

The loss of DNA methylation is a common phenomenon in human tumor genomes [9, 47, 48]. This molecular change occurs primarily in the genome, intergenic region, and repetitive DNA elements [10]. It is thought to be associated with chromosomal instability, reactivation of transposable factors, and loss of genomic imprinting [11]. However, little is known about the effects of DNA hypomethylation on tumors, and a possible hypothesis is that it leads to oncogenes' transcriptional activation [13, 49]. We determined whether LOX methylation status will affect LOX mRNA expression in ESCA through Pearson coefficients. LOX mRNA expression was significantly negatively correlated with LOX methylation in ESCA tissues (*r* = −0.3609, *p* < 0.0001). Then, we further found that LOX methylation levels were significantly associated with several clinicopathological parameters. Besides, the prognostic significance of DNA methylation at different sites of LOX was also investigated. Nevertheless, we found a paradox: methylation at different sites shows different effects on survival. Frankly, the evidence that hypomethylation activates oncogenes is still ambiguous. On the one hand, well-known oncogenes are hypomethylated in tumors [50, 51], but this is not convincingly related to their transcriptional activation [52, 53]. On the other hand, genes activated by promoter hypomethylation in tumors have been identified [12]. Therefore, the exact effects of methylation at different LOX gene sites on expression and function require further experimental verification.

Many researchers recognized that extracellular matrix (ECM) and its recombination are essential to the formation of the immune microenvironment and the evolution of tumors [54]. LOX is a crucial factor in the dynamic balance of ECM; therefore, it is not difficult to infer that its dysregulation will impact the TIME. However, it is not clear how the LOX regulates TIME. Our study showed that LOX expression in ESCA was significantly associated with the immune subtype; besides, LOX expression and TMB showed a completely different relationship in the different immune subtypes. Furthermore, the expression of ICPs differed significantly between different immune subtypes. TMB and PD-L1 are helpful markers for predicting the effectiveness of ICP blockade (ICB) in specific cancer types [55]. Hence, LOX may be an effective marker for predicting the therapeutic effect of ICP blockade therapy. Immune cells are part of the TME and are involved in the biological behavior and survival of ESCA patients [16]. From the TCGA and the GEO database analysis, LOX was significantly associated with the level of macrophage infiltration, which is commonly referred to as tumor-associated macrophages infiltrating the tumor stroma [35]. The macrophage colony-stimulating factor (M-CSF), C-C motif chemokine ligand 2 (CCL2), and vascular endothelial growth factor (VEGF) specifically recruit circulating monocytes and ultimately convert them into TAM [35]. Under LPS or IFN-*γ* stimulation, macrophages differentiate into M1 (classical activation); by interleukin stimulation, such as IL-4, IL-10, and IL-13, high expressions of immunosuppressive molecules, such as CD206, macrophages differentiate into M2 [34, 35, 56]. From the current study, we found that LOX expression was positively related to macrophage infiltration and expression of M1 and M2 markers in ESCA tissues, but only the factors secreted by M2-type macrophages, which were mainly related to the promotion of the formation of the immunosuppressive environment (IL10, CCL2), angiogenesis (PDGFB), tumor proliferation (ARG1), and accelerating tumor recurrence and metastasis (EGF, TGF-*β*, CCL22, MMP2, MMP9, and MMP14), were significantly positively correlated with the expression of LOX [56]. A previous analysis report showed that in more than 80% of the studies, TAM infiltration is often considered a protumorigenic factor associated with dismal prognosis [57]. Our results were consistent with these studies. Besides, we also found that low LOX expression and low-level infiltration of macrophages have shown better survival. The current breakthrough in using TAM as an oncology research strategy involves reducing TAM in tumors and transforming M2-type macrophages into M1 phenotypes [58]. From our study, LOX is possibly an effective therapeutic target to reduce TAM infiltration and inhibit the function of M2 in ESCA. These results emphasize the importance of LOX in ESCA development. Therefore, targeting the LOX can take advantage of the development fragility of ESCA and should be regarded as a feasible treatment strategy.

There are some limitations. Our research showed that LOX is hypomethylated and highly expressed in ESCA, but the specific relationship between the two remains further confirmed. Secondly, the specific mechanism of LOX regulating TAM needs further study, such as the levels of TAM infiltration and the secretion of M2 TAM cytokines. Thirdly, although we conducted preliminary validation in clinical samples, more detailed investigations on the molecular mechanisms of LOX in ESCA remain to be explored.

## Data Availability

The original data supporting the conclusions of this manuscript are made available by the author to any qualified researcher without undue preservation.

## Supplementary Materials

Supplementary Materials Figure S1: Differential expression genes and related biological pathways in the TCGA-ESCA database. (A) Volcano plot showing the distribution of the adjusted *p* values and fold changes, with red dots representing overexpressed mRNAs and blue dots representing statistically significant underexpressed mRNAs. (B) Hierarchical clustering analysis of DEGs between tumor tissues (*N* = 162) and normal tissues (*N* = 1456). KEGG pathway enrichment analysis of upregulated DEGs (C) and downregulated DEGs (E). GO biological process enrichment analysis of upregulated DEGs (D) and downregulated DEGs (F). Figure S2: Correlation between IFI44 mRNA, IL18 mRNA, and SLURP1mRNA expression and OS. (A) DEG PPI network was created with Cytoscape. (B) IFI mRNA is highly expressed in ESCA tissues from TCGA datasets. (C) IL18 mRNA is lowly expressed in ESCA tissues from TCGA datasets. (D) SLURP1 mRNA is lowly expressed in ESCA tissues from TCGA datasets. (E) Kaplan–Meier curves of low and high IFI expression in ESCA patients. (F) Kaplan–Meier curves of low and high IL18 expression in ESCA patients. (G) Kaplan–Meier curves of low and high SLURP1 expression in ESCA patients. Figure S3: KEGG and GO pathway enrichment analysis of LOX-related biological pathways in ESCA. KEGG pathway enrichment analysis of all LOX-related genes (A). Upregulated LOX-related genes (C); downregulated LOX-related genes (E). Gene Ontology (GO) biological process enrichment analysis of all LOX-related genes (B); upregulated LOX-related genes; (D) and downregulated LOX-related genes (F). The dot size and color intensity represent the gene count and enrichment level, respectively. Figure S4: Kaplan–Meier curves of low and high LOX DNA methylation at different sites in ESCA patients. (A) Cg05256605. (B) Cg09262269. (C) Cg22836153. (D) Cg23352712. (E) Cg02548238. (F) Cg08431704. (G) Cg01824804. (H) Cg01429231. (I) Cg09499414. Figure S5: Association between LOX DNA methylation and clinicopathological parameters of ESCA patients' LOX DNA methylation level stratified by histology type (A); gender (B); age (C); race (D); alcohol consumption frequencies (E); person cigarette smoking history pack-year (F); tumor stage (G); lymph node stage (H); metastasis stage (I); and disease-free status (J). Each point represents a patient. Figure S6: Expression of LOX mRNA and TAM-related soluble factors mRNA expression. (A) Soluble factors secreted by M2: TGFB, EGF, CCL22, MMP2, MMP9, MMP14, TNF, and IL6. (B) M1 polarization inducing factor, IFN-*γ*, M2 polarization inducing factor IL4, IL13, and IL10. Figure S7: Kaplan–Meier curves of macrophage infiltration and LOX expression in ESCA patients. (A) Low macrophage infiltration and high macrophage infiltration estimated by EPIC. (B) Low macrophage infiltration and low LOX expression, low macrophage infiltration and high LOX expression, high macrophage infiltration and low LOX, and high macrophage infiltration and high LOX estimated by EPIC. (C) Low macrophage infiltration and high macrophage infiltration estimated by XCELL. (D) Low macrophage infiltration and low LOX expression, low macrophage infiltration and high LOX expression, high macrophage infiltration and low LOX, and high macrophage infiltration and high LOX estimated by XCELL. (E) Low M2 type macrophage infiltration and high M2 type macrophage infiltration estimated by XCELL. (F) Low M2 type macrophage infiltration and low LOX expression, low M2 type macrophage infiltration and high LOX expression, high M2 type macrophage infiltration and low LOX, and high M2 type macrophage infiltration and high LOX estimated by XCELL. Table 1: Differential genes in 4 GEO-ESCA cohorts. Table 2: Overlap differential genes in 4 GEO-ESCA cohorts. Table 3: Website tools in method. Table 4: Correlation between LOX expression, methylation, and clinicopathological features in TCGA.
