# Supplementary material for: LOX and Its Methylation Impact Prognosis of Diseases and Correlate with TAM Infiltration in ESCA
Source: J Oncol. 2022 Aug 31;2022:5111237. doi: 10.1155/2022/5111237 (PMC9452977; doi:10.1155/2022/5111237)
Supplement: Supplementary Materials — Figure S1: Differential expression genes and related biological pathways in the TCGA-ESCA database. (A) Volcano plot showing the distribution of the adjusted p values and fold changes, with red dots representing overexpressed mRNAs and blue dots representing statistically significant underexpressed mRNAs. (B) Hierarchical clustering analysis of DEGs between tumor tissues (N = 162) and normal tissues (N = 1456). KEGG pathway enrichment analysis of upregulated DEGs (C) and downregulated DEGs (E). GO biological process enrichment analysis of upregulated DEGs (D) and downregulated DEGs (F). Figure S2: Correlation between IFI44 mRNA, IL18 mRNA, and SLURP1mRNA expression and OS. (A) DEG PPI network was created with Cytoscape. (B) IFI mRNA is highly expressed in ESCA tissues from TCGA datasets. (C) IL18 mRNA is lowly expressed in ESCA tissues from TCGA datasets. (D) SLURP1 mRNA is lowly expressed in ESCA tissues from TCGA datasets. (E) Kaplan–Meier curves of low and high IFI expression in ESCA patients. (F) Kaplan–Meier curves of low and high IL18 expression in ESCA patients. (G) Kaplan–Meier curves of low and high SLURP1 expression in ESCA patients. Figure S3: KEGG and GO pathway enrichment analysis of LOX-related biological pathways in ESCA. KEGG pathway enrichment analysis of all LOX-related genes (A). Upregulated LOX-related genes (C); downregulated LOX-related genes (E). Gene Ontology (GO) biological process enrichment analysis of all LOX-related genes (B); upregulated LOX-related genes; (D) and downregulated LOX-related genes (F). The dot size and color intensity represent the gene count and enrichment level, respectively. Figure S4: Kaplan–Meier curves of low and high LOX DNA methylation at different sites in ESCA patients. (A) Cg05256605. (B) Cg09262269. (C) Cg22836153. (D) Cg23352712. (E) Cg02548238. (F) Cg08431704. (G) Cg01824804. (H) Cg01429231. (I) Cg09499414. Figure S5: Association between LOX DNA methylation and clinicopathological parameters of ESCA patients [file 5111237.f1.zip › Supplementary Tables-table 4.pdf]

Clinicopathological characteristics of patients in TCGA

Catagory	TCGA-Expression N	%	Pvalue	TCGA-Methylation N	%	Pvalue
**Gender**						
Male	137	85.63		159	85.48	
Female	23	14.37	p=0.9390	27	14.52	p=0.2739
**Age**						
<60	93	50. 27		92	50.00	
>60	92	49.73	p=0.1540	92	50.00	p=0.0105
**Histology**						
EAC	91	53.53		89	47.85	
ESCC	79	46.47	p<0.001	97	52.15	p<0.001
**Race**						
Asian	46	27.88		46	27.71	
Black or African American	6	3.64	p=0.25	6	3.61	p=0.7060
White	113	68.48	p<0.0001	114	68.68	p=0.0034
**Drinking (Times/Week)**						
0	35	30.70		35	30.70	
1~3	23	20.18	p=0.0311	23	20.18	p=0.0062
4~7	56	49.12	p=0.0193	56	49.12	p=0.0023
**Smoking (Pack/year)**						
<30	50	51.02		50	51.02	
>30	48	48.98	p=0.2767	48	48.98	p=0.4527
**Tumor stage**						
T1	31	18.56		31	18.56	
T2	43	25.75	p<0.0001	43	25.75	p=0.0005
T3	88	52.69	p=0.0011	88	52.69	p=0.0090
T4	5	3.00	p=0.0027	5	3.00	p=0.1610
**Lymph Node**						
Nx	14	21.21		14	21.21	
N0	26	39.39		26	39.39	
N1	23	34.85	p=0.8973	23	34.85	p=0.5938
N2	3	4.55	p=0.4900	3	4.55	p=0.6125
**Metastasis**						
Mx	18	11.04		18	11.04	
M0	136	83.44		136	83.44	
M1	9	5.52	p=0.4540	9	5.52	p=0.9647
**Disease statu**						
DiseaseFree	71	49.65		71	49.65	
Recurred/Progressed	72	50.35	p=0.7429	72	50.35	p=0.7727
