# Supplementary material for: LOX and Its Methylation Impact Prognosis of Diseases and Correlate with TAM Infiltration in ESCA
Source: J Oncol. 2022 Aug 31;2022:5111237. doi: 10.1155/2022/5111237 (PMC9452977; doi:10.1155/2022/5111237)
Supplement: Supplementary Materials — Figure S1: Differential expression genes and related biological pathways in the TCGA-ESCA database. (A) Volcano plot showing the distribution of the adjusted p values and fold changes, with red dots representing overexpressed mRNAs and blue dots representing statistically significant underexpressed mRNAs. (B) Hierarchical clustering analysis of DEGs between tumor tissues (N = 162) and normal tissues (N = 1456). KEGG pathway enrichment analysis of upregulated DEGs (C) and downregulated DEGs (E). GO biological process enrichment analysis of upregulated DEGs (D) and downregulated DEGs (F). Figure S2: Correlation between IFI44 mRNA, IL18 mRNA, and SLURP1mRNA expression and OS. (A) DEG PPI network was created with Cytoscape. (B) IFI mRNA is highly expressed in ESCA tissues from TCGA datasets. (C) IL18 mRNA is lowly expressed in ESCA tissues from TCGA datasets. (D) SLURP1 mRNA is lowly expressed in ESCA tissues from TCGA datasets. (E) Kaplan–Meier curves of low and high IFI expression in ESCA patients. (F) Kaplan–Meier curves of low and high IL18 expression in ESCA patients. (G) Kaplan–Meier curves of low and high SLURP1 expression in ESCA patients. Figure S3: KEGG and GO pathway enrichment analysis of LOX-related biological pathways in ESCA. KEGG pathway enrichment analysis of all LOX-related genes (A). Upregulated LOX-related genes (C); downregulated LOX-related genes (E). Gene Ontology (GO) biological process enrichment analysis of all LOX-related genes (B); upregulated LOX-related genes; (D) and downregulated LOX-related genes (F). The dot size and color intensity represent the gene count and enrichment level, respectively. Figure S4: Kaplan–Meier curves of low and high LOX DNA methylation at different sites in ESCA patients. (A) Cg05256605. (B) Cg09262269. (C) Cg22836153. (D) Cg23352712. (E) Cg02548238. (F) Cg08431704. (G) Cg01824804. (H) Cg01429231. (I) Cg09499414. Figure S5: Association between LOX DNA methylation and clinicopathological parameters of ESCA patients [file 5111237.f1.zip › Supplementary Tables-Table1.docx]

**Table 1: differential genes in 4 GEO-ESCA cohort**

| **GSE20347** | **GSE67269** | **GSE23400** | **GSE38129** |
| --- | --- | --- | --- |
| CRISP3 | CRISP3 | MMP1 | CRISP3 |
| CRNN | MMP1 | CRISP3 | MMP1 |
| MAL | CRNN | MAL | CRNN |
| CRCT1 | MAL | CRNN | SPP1 |
| TGM3 | TGM3 | SCEL | MAL |
| SCEL | CLCA4 | CLCA4 | COL11A1 |
| CLCA4 | SPP1 | TGM3 | CRCT1 |
| CLIC3 | CRCT1 | SPP1 | TGM3 |
| KRT4 | SCEL | CRCT1 | ADH1B |
| MMP1 | COL11A1 | TMPRSS11E | CLCA4 |
| SLURP1 | SLURP1 | POSTN | SLURP1 |
| SPINK5 | FLG | COL1A1 | SCEL |
| SPP1 | KRT4 | SLURP1 | CLIC3 |
| TMPRSS11E | TMPRSS11E | CLIC3 | MMP12 |
| ENDOU | SPINK5 | JUP /// KRT17 | HPGD |
| HPGD | MMP12 | KLK13 | ENDOU |
| COL11A1 | CLIC3 | ENDOU | FLG |
| KLK13 | KLK13 | IL1RN | COL10A1 |
| CEACAM7 | ENDOU | SPINK5 | JUP /// KRT17 |
| FLG | HPGD | PPP1R3C | KRT4 |
| EREG | MAGEA3 /// MAGEA6 | COL1A2 | POSTN |
| CXCR2 | COL1A1 | HPGD | SPINK5 |
| PPP1R3C | MAGEA6 | RHCG | NTS |
| KRT13 | POSTN | KRT4 | PPP1R3C |
| CYP4B1 | ADH1B | COL11A1 | CYP4B1 |
| EPB41L3 | PPP1R3C | FLG | TMPRSS11E |
| RHCG | ECM1 | CDH11 | CXCR2 |
| CEACAM5 | CEACAM7 | ECM1 | KLK13 |
| SERPINB2 | CXCR2 | EMP1 | CEACAM7 |
| CEACAM6 | COL10A1 | KRT13 | GPX3 |
| NTS | GPX3 | CEACAM6 | CST1 |
| EMP1 | COL1A2 | ADH1B | COL1A1 |
| FMO2 | PSCA | PSCA | EREG |
| HOPX | EREG | MMP12 | EPB41L3 |
| ECM1 | CYP4B1 | HOPX | COL1A2 |
| POSTN | KRT13 | MAGEA6 | EMP1 |
| ANO1 | IL1RN | PTHLH | APOBEC3B |
| MMP12 | SERPINB2 | MAGEA3 /// MAGEA6 | ECM1 |
| TFAP2B | RHCG | SNAI2 | MMP10 |
| SERPINB1 | JUP /// KRT17 | VCAN | FMO2 |
| SERPINB3 | CEACAM5 | ALOX12 | PSCA |
| GPX3 | EMP1 | MMP10 | CXCL8 |
| IL1RN | GYS2 | COL3A1 | MAGEA3 /// MAGEA6 |
| SERPINB13 | FMO2 | CEACAM7 | MAGEA6 |
| CWH43 | SPRR3 | GPX3 | MGLL |
| SPRR3 | UPK1A | EPS8L1 | HOXB7 |
| TGM1 | CST1 | SULF1 | CITED2 |
| ACPP | SPRR2C | ECT2 | SULF1 |
| VCAN | ACPP | MUC5B | VCAN |
| SERPINB4 | MMP10 | FUT6 | SERPINB2 |
| BBOX1 | ALOX12 | COL5A2 | PTHLH |
| DSG1 | PPL | TGM1 | SORBS2 |
| BLNK | TFAP2B | CFD | KAT2B |
| CXCL8 | CEACAM6 | TMPRSS2 | ANO1 |
| SLC16A7 | TGM1 | SERPINB13 | CWH43 |
| SPRR2C | VCAN | PPL | TFAP2B |
| PSCA | CWH43 | SERPINB1 | ID4 |
| IL18 | APOBEC3B | CEACAM5 | GYS2 |
| CD24 | CITED2 | FUT3 | SLC16A7 |
| MGLL | CXCL8 | SERPINB2 | BBOX1 |
| MALL | CDH11 | MYH11 | CDH11 |
| COL1A2 | EPB41L3 | TOP2A | IL1RN |
| PTK6 | SORBS2 | BLNK | INHBA |
| COL1A1 | ANXA9 | PLAU | ABCA8 |
| PLAC8 | PRSS3 | NUCB2 | KRT13 |
| CRABP2 | SULF1 | GABRP | LAMC2 |
| SERPINB3 | SPRR2B | PRSS3 | CEACAM5 |
| C1orf116 | MMP3 | CCNG2 | TGM1 |
| JUP /// KRT17 | MMP13 | CKS2 | ECT2 |
| KAT2B | IL18 | FMO2 | ZIC1 |
| CYP3A5 | COL3A1 | SPRR2C | S100P |
| MMP10 | CYP2C18 | TMPRSS11D | IGHG1 |
| AKR1C1 | SCNN1B | ZNF185 | MMP13 |
| PPL | IGHG1 | ADIRF | RHCG |
| ZNF185 | HOXB7 | INHBA | MMP3 |
| GYS2 | INHBA | SERPINB4 | EPCAM |
| BEX4 | MGLL | SERPINB3 | FCER1A |
| CEACAM1 | TMPRSS2 | IL36A | HOXA9 |
| ALOX12 | SERPINB1 | LPIN1 | HSPB8 |
| SULF1 | BBOX1 | ISG15 | SERPINB1 |
| UPK1A | APOC1 | DUSP5 | CEACAM6 |
| ZIC1 | C1orf116 | CRABP2 | BUB1 |
| PTN | SYNPO2L | CEP55 | AKR1C1 |
| CYP2C18 | C2orf54 | SPRR2B | SPRR2C |
| EHF | EPS8L1 | TGFBI | TOP2A |
| CITED2 | CYP3A5 | NMU | PLAC8 |
| APOBEC3B | SERPINB13 | MXD1 | TMPRSS2 |
| C2orf54 | CEACAM1 | C1orf116 | COL3A1 |
| TTC9 | ZNF185 | CLEC3B /// EXOSC7 | ACPP |
| FCER1A | MALL | CD24 | IL18 |
| EPS8L1 | HOPX | EPCAM | HOXA10 |
| KLK12 | CD24 | LAMB3 | CEP55 |
| ID4 | HEY1 | KAT2B | HEY1 |
| SCNN1B | KLK12 | PITX1 | PRSS3 |
| GALNT12 | HOXA9 | COL5A1 | ANXA9 |
| S100P | GDPD3 | SOX4 | BLNK |
| EPHX3 | CCNG2 | SPARC | MEST |
| NEBL | COL5A2 | TNC | MFAP2 |
| SPRR2B | EPHX3 | CST1 | MMP11 |
| DIO2 | PLAC8 | CXCL8 | UPK1A |
| ANXA9 | DUSP5 | SERPINB3 | UCHL1 |
| CDA | LAMC2 | MALL | CYP3A5 |
| ADH1B | ECT2 | LAMC2 | PPL |
| CDH11 | TTC9 | IL18 | AURKA |
| PRSS3 | PLAU | ABLIM1 | PLAU |
| INHBA | ANO1 | EHD3 | IGF2BP3 |
| GDPD3 | TOP2A | RFC4 | COL5A2 |
| UBL3 | MXD1 | EHF | DLGAP5 |
| CH25H | MFAP2 | CDH3 | KRT24 |
| SULT2B1 | MMP11 | IGFBP3 | KIF4A |
| IGHG1 | MEST | S100P | C1orf116 |
| GABRP | HOXA10 | PLAC8 | CXCL12 |
| PTHLH | SERPINB3 | KLK12 | PTN |
| MAGEA3 /// MAGEA6 | SULT2B1 | MFAP2 | EPHX3 |
| TMPRSS2 | ID4 | SPRR3 | GDPD3 |
| SLC24A3 | TMPRSS11D | SASH1 | KIF23 |
| DUSP5 | ZNF365 | ASPM | PPFIA1 |
| UCHL1 | PTK6 | CYP4B1 | KLK12 |
| CCNG2 | KAT2B | TTC9 | DSG1 |
| TMPRSS11D | CXCL10 | RANBP9 | BEX4 |
| KLK11 | CFD | SCNN1B | SCNN1B |
| ZNF750 | ABCA8 | ODC1 | CH25H |
| SPRR1A | IGFBP3 | HSPB8 | CYP2C18 |
| NUCB2 | PTHLH | LAPTM4B | UBL3 |
| MAGEA6 | BLNK | MGLL | GALNT12 |
| IVL | S100P | PRC1 | CDK1 |
| SLPI | HSPB8 | SULT2B1 | MALL |
| TP53I3 | FCER1A | FSCN1 | DUSP5 |
| EPCAM | SNX10 | UPK1A | EPS8L1 |
| EXPH5 | ADIRF | ID4 | NEK2 |
| ISG15 | TGFBI | MMP11 | TRIP13 |
| HOXB7 | FUT6 | CEACAM1 | CEACAM1 |
| ABLIM1 | GABRP | CYP3A5 | HOPX |
| COL10A1 | ISG15 | TP63 | ALOX12 |
| SIM2 | CEP55 | FZD6 | CFD |
| ERO1L | SFRP4 | UBL3 | ADIRF |
| SASH1 | KLK11 | GLTP | SPRR3 |
| COL5A2 | CLEC3B /// EXOSC7 | OBFC1 | ATAD2 |
| ECT2 | CDA | KLK11 | CENPA /// SLC35F6 |
| MPZL2 | BUB1 | AIM1L | TTC9 |
| ODC1 | C18orf25 | LCN2 | C7 |
| OBFC1 | ABLIM1 | CXCL12 | TGFBI |
| HEY1 | ZIC1 | HMGB3 | KIF14 |
| MARCKSL1 | EPCAM | SCNN1A | C2orf54 |
| ANXA3 | LCN2 | GINS1 | PBK |
| TGFBI | ATAD2 | CDC20 | SNX10 |
| EHD3 | DLGAP5 | RRM2 | ABLIM1 |
| SLC39A14 | MARCKSL1 | CDK1 | SERPINE1 |
| DSC2 | CYP2E1 | ITGA6 | APOC1 |
| ACKR3 | NTS | NUSAP1 | CD24 |
| AQP3 | SPARC | MEST | RAD51AP1 |
| MXD1 | MMP9 | SERPINE1 | MARCKSL1 |
| HSPB8 | SERPINE1 | FOXM1 | CLEC3B /// EXOSC7 |
| TMOD3 | ABLIM3 | CKS1B | SOX4 |
| EVPL | HLF | EREG | NUCB2 |
| RRAGD | SASH1 | LAMA3 | HLF |
| PALMD | HOXC10 | THBS2 | IGFBP3 |
| HS3ST1 | AURKA | DTL | UBE2C |
| IGFBP3 | SERPINB4 | SIM2 | TFRC |
| LYPD3 | UBL3 | COL7A1 | ISG15 |
| SLC16A6 | NEK2 | EPB41L3 | MET |
| MMP13 | C7 | TMEM45A | TPX2 |
| PITX1 | PTN | IGF2BP2 | CALB1 |
| DHRS1 | IVL | ASPN | PTK6 |
| FUT3 | KIF4A | DIO2 | ITM2A |
| AIM1L | DSG1 | IGH /// IGHA1 /// IGHA2 | CCNG2 |
| COL3A1 | CDK1 | PAX9 | SERPINB13 |
| LCN2 | EXPH5 | CRYAB | MAGEA11 |
| NMU | SERPINB3 | BUB1B | CDH3 |
| RANBP9 | KIF20A | SERPINH1 | NELL2 |
| MEST | MYH11 | ZNF365 | SPARC |
| KRT24 | BEX4 | AKR1C1 | SASH1 |
| VAV3 | GALNT12 | C2orf54 | GABRP |
| S100A9 | SPRR1A | APOE | LAMB3 |
| IGF2BP3 | NELL2 | UPK3B | CENPN |
| CAMK2N1 | GPD1L | BGN | SULT2B1 |
| PPFIA1 | FUT3 | MMP9 | TCN1 |
| LUM | RRAD | TTK | EHF |
| DUOX1 | CENPA /// SLC35F6 | SORBS1 | MXD1 |
| LAMC2 | HMGA2 | ZNF750 | RRM2 |
| MFAP2 | LAMP3 | MET | RFC4 |
| PFN2 | LAMB3 | PHLDA1 | BIRC5 |
| ITPR2 | CXCL12 | CALB1 | CDKN3 |
| HMGA2 | LOX | PTK6 | BID |
| TJP1 | UBE2C | CITED2 | GPD1L |
| HOXA9 | ADH7 | COL6A3 | CDC20 |
| SPARC | MET | CYP2C18 | DTL |
| ADH7 | OBFC1 | CCNB1 | FHL1 |
| HPSE | CXCL13 | GMDS | COL14A1 |
| FADS1 /// MIR1908 | RAD51AP1 | EPS8L2 | THBS2 |
| SEMA3C | SOX4 | MCM2 | FANCI |
| MET | BID | DUOX1 | SLC7A11 |
| LAPTM4B | FOXM1 | TP53I3 | FSCN1 |
| RIOK3 | PCP4 | MTHFD2 | ABLIM3 |
| EPS8L2 | CRYAB | NETO2 | ASPM |
| COL4A2 | GINS1 | NDRG2 | FOS |
| GMDS | CRABP2 | RAB11FIP1 | ZNF185 |
| GPD1L | CH25H | TNXA /// TNXB | MYH11 |
| MFHAS1 | EVPL | HEY1 | MCM2 |
| CES2 | TPX2 | DLGAP5 | HMGA2 |
| FUT6 | SIM2 | AQP3 | MAD2L1 |
| SCNN1A | DTL | EVPL | PRC1 |
| AHNAK | TRIP13 | PBK | CXCL1 |
| COL4A1 | TCN1 | SPRR1A | COBL |
| OR7E14P | TJP1 | KIF4A | KIF20A |
| ZNF365 | PBK | BIRC5 | OBFC1 |
| TTC39A | GLTP | LUM | KLK11 |
| RAB25 | UCHL1 | CES2 | RBPMS |
| TNFAIP6 | KRT24 | PDZD2 | LAMP3 |
| FN1 | CDKN3 | SLC39A6 | GINS1 |
| IGF2BP2 | KIF14 | ABCA8 | LAPTM4B |
| FNDC3B | S100A12 | CNN1 | CDC6 |
| AIM1 | CDH3 | ANO1 | CPEB3 |
| SNX10 | MAGEA11 | FNDC3B | SYNPO2L |
| S100A14 | EHD3 | ABLIM3 | FOXM1 |
| CPEB3 | LAPTM4B | BEX4 | CRABP2 |
| CYP2E1 | RFC4 | CWH43 | CRYAB |
| C18orf25 | SERPINH1 | PAICS | TMPRSS11D |
| PI3 | SLC16A7 | KRT24 | CAMK2N1 |
| LIMCH1 | NUCB2 | PDLIM2 | NDC80 |
| IL13RA1 | ASPN | SMC2 | SNAI2 |
| BUB1 | AHNAK | TFRC | SPC25 |
| AURKA | ANXA3 | PCP4 | HMMR |
| HOXA10 | THBS2 | TMOD3 | CCNB2 |
| SOX4 | FAP | MCM4 | KIF2C |
| DNASE1L3 | AIM2 | GDPD3 | KLF4 |
| CST1 | TNXA /// TNXB | YOD1 | CENPF |
| CST6 | KANK1 | KANK1 | CYP2E1 |
| DPYD | COL5A1 | SORBS2 | KANK1 |
| CNN3 | IL36A | FST | CDA |
| PLAU | CES2 | EPHX3 | BUB1B |
| KIF23 | ATP1A2 | MARCKSL1 | TNFAIP6 |
| SORBS2 | PITX1 | FHL1 | SLPI |
| SMAGP | CDC20 | PLXNA1 | NEBL |
| RFC4 | RORA | RMND5B | SIM2 |
| DDAH1 | RANBP9 | RAI14 | TNXA /// TNXB |
| HLF | FSCN1 | KRT14 | RPL39L |
| LY6D | CXCL1 | GPNMB | SLC24A3 |
| IFI44L | RIOK3 | PFN2 | HOXC10 |
| KANK1 | AQP3 | RCAN2 | IVL |
| CRYAB | AKR1C1 | HLF | TTK |
| ELOVL4 | ERO1L | LOXL2 | HBA1 /// HBA2 |
| NELL2 | HMMR | GMNN | PLN |
| SORT1 | CXCL9 | NTRK2 | IGK /// IGKC |
| SAMD9 | SLPI | UBE2C | RRAD |
| ZDHHC13 | ASPM | CDKN3 | IGF2BP2 |
| KIF14 | BIRC5 | PRH1-PRR4 /// PRR4 | MCM10 |
| CAST | DUOX1 | GPD1L | CXCL10 |
| MUC1 | CENPF | KIF14 | FUT6 |
| KLK10 | MAD2L1 | LMOD1 | EXPH5 |
| RBPMS | SNAI2 | ALDH3A1 | CKS1B |
| CRIP2 | UPK3B | GYS2 | SFRP4 |
| RORA | APOE | RBM47 | AURKB |
| ABLIM3 | MCM2 | PLAGL1 | CHRDL1 |
| ELOVL6 | ZNF750 | MMP13 | LCN2 |
| ADIRF | IFI6 | NEK2 | P2RY14 |
| GULP1 | PPFIA1 | SLC25A32 | SLC16A6 |
| TRIP10 | DHRS1 | DPT | PFN2 |
| AREG | DNASE1L3 | LDB3 | EVPL |
| ALDH3B2 | RRM2 | ITM2A | AQP3 |
| S100A12 | MT1M | SLPI | GINS2 |
| RAD51AP1 | LDB3 | TRIP10 | ODC1 |
| MIR4680 /// PDCD4 | MAGED4 | STK39 | CKS2 |
| MCM2 | PLA2G7 | IGHG1 | MAGED4 |
| TFRC | DIO2 | TJP1 | LIMCH1 |
| SNAI2 | KIF23 | MCM6 | PALMD |
| GLTP | AIM1L | TUSC3 | DHRS1 |
| ABHD5 | ADAM12 | DSG2 | SERPINH1 |
| ABAT | CYP2J2 | TPX2 | PDZD2 |
| FOS | LPIN1 | NEFL | KIAA0101 |
| THBS2 | TMOD3 | CENPF | MTHFD2 |
| PDLIM2 | NEBL | PRKDC | FST |
| ATAD2 | NMU | ERO1L | CRIP2 |
| PHLDA1 | ZNF426 | S100A14 | TP53I3 |
| KLF4 | COBL | AURKA | DNASE1L3 |
| CEP55 | PRC1 | U2SURP | STIL |
| CYP2J2 | KIAA0101 | PCNA | ZNF365 |
| BID | FANCI | BASP1 | DIO2 |
| TCN1 | RAB11FIP1 | ACPP | ATP1A2 |
| SERPINE1 | LOXL2 | STAT1 | ANXA3 |
| COBL | CXCL11 | KIAA0232 | EHD3 |
| LPCAT1 | CPEB3 | MELK | MPZL2 |
| KCNS3 | PDZD2 | CBX3 | GCLM |
| KIF4A | IGK /// IGKC | SLC16A1 | NUSAP1 |
| RAB11FIP1 | OR7E14P | METTL7A | ABAT |
| FRMD4B | FNDC3B | ENAH | CTTN |
| MMP2 | ALDH3A1 | FAP | RORA |
| ENAH | CKS2 | CXCR2 | SORBS1 |
| DLGAP5 | BGN | KIAA0101 | ITGA8 |
| CENPA /// SLC35F6 | TP53I3 | TMEM97 | ITGA6 |
| MAGEA11 | PRSS2 | VOPP1 | ITPR2 |
| CDKN3 | SORT1 | ALCAM | PLAUR |
| RBM47 | SMAGP | COL10A1 | LRP12 |
| ATP10B | LYPD3 | MAD2L1 | NETO2 |
| GPR126 | IFI44L | MAFF | FUT3 |
| DHRS9 | MPZL2 | HLTF | TMOD3 |
| DYNLT3 | COL6A3 | ACTL6A | NRCAM |
| SERPINH1 | HMGB3 | AIM1 | HS3ST1 |
| RBP1 | MAGEA12 | KRT15 | DPT |
| COL6A3 | PLAUR | MMP3 | GTSE1 |
| PDZK1IP1 | MCM10 | MCM5 | ERO1L |
| TRIP13 | FHL1 | CAST | C18orf25 |
| IGLC1 | DPT | FAT1 | MMP9 |
| HLTF | RPL39L | ACTG2 | RANBP9 |
| LGALS1 | CST6 | CDA | FNDC3B |
| CXCL10 | GTSE1 | DST | PCP4 |
| KLK8 | KIF2C | LAMP3 | FAP |
| KLK7 | ADAMDEC1 | ELF3 | CCNB1 |
| COL5A1 | IGF2BP2 | MYO1B | MAOB |
| LOXL2 | TRIP10 | MYO6 | PLP1 |
| TXNRD1 | MYOC | MIR4680 /// PDCD4 | COL6A3 |
| CALB1 | CAMK2N1 | MUC1 | PITX1 |
| RRAD | EHF | ECHDC2 | DUSP1 |
| SPAG16 | EDN3 | RIOK3 | CCNA1 |
| MTHFD2 | KLF4 | ANXA9 | TP63 |
| SYNPO2L | BUB1B | ATAD2 | TMEM100 |
| MAGED4 | TNFAIP6 | ZWINT | COL5A1 |
| TLR3 | TTK | PTPRZ1 | ACKR1 |
| PKP3 | SLC16A6 | TYMS | HMGB3 |
| IQCJ-SCHIP1 /// SCHIP1 | NDC80 | IGF2BP3 | HBB |
| PXDN | TFRC | SLC7A11 | TGFBR3 |
| MANSC1 | RARRES1 | KLF4 | MELK |
| CDK1 | ABHD5 | HSPD1 | RRAGD |
| LGALS7 /// LGALS7B | CENPN | CTSC | FAM107A |
| FST | RBPMS | HAT1 | DNMT3B |
| SLC7A11 | KLK7 | HOMER3 | TRIP10 |
| ZNF426 | IGLC1 | MYL9 | LOXL2 |
| ENO2 | PHLDA1 | GCLM | IL33 |
| MLF1 | FAM107A | ITGB4 | OR7E14P |
| LRP12 | CCNB2 | GREM1 | CCDC69 |
| ITGA6 | RCAN2 | PXDN | WDHD1 |
| TOP2A | NUSAP1 | PHACTR2 | DUOX1 |
| CTTN | ITGA8 | SFRP4 | BLM |
| ETFDH | RRAGD | ZDHHC13 | ATP10B |
| SPRR1B | HS3ST1 | HSPH1 | ETFDH |
| C1S | NETO2 | TGFBR3 | PAIP2B |
| THBS1 | CKS1B | GALNT12 | BGN |
| IGK /// IGKC | BAMBI | NEBL | DPYD |
| RNF141 | PAX9 | SMAGP | SLC39A14 |
| LY96 | FOS | FADS1 /// MIR1908 | EDN3 |
| EYA2 | FOSB | SLC39A14 | TTC39A |
| ALDH3A1 | CDC6 | HOXA9 | XK |
| PBK | CYP4F3 | PTN | RCAN2 |
| NEK2 | AURKB | TRIP13 | MYO5A |
| FGFBP1 | SLC24A3 | EXPH5 | CES2 |
| ACOX3 | SCNN1A | CXCL1 | GGH |
| MMP3 | TDO2 | DES | CCL14 /// CCL15-CCL14 |
| GCHFR | MELK | PMEPA1 | SLC2A1 |
| CKS1B | MIR4680 /// PDCD4 | AHNAK | DSG2 |
| ACOX1 | TTC39A | ELOVL6 | GMDS |
| VOPP1 | PAIP2B | RBPMS | LUM |
| ALCAM | PMEPA1 | PADI1 | ZNF426 |
| RPL39L | ANXA1 | FOSB | KCNS3 |
| COL14A1 | CALB1 | RAB25 | METTL7A |
| DOPEY2 | ETFDH | TIMP1 | ORC6 |
| ITM2A | CRIP2 | SLK | CCNE2 |
| MSANTD3-TMEFF1 /// TMEFF1 | BLM | PPFIA1 | FN1 |
| PHACTR2 | ABAT | HPRT1 | DDAH1 |
| SH3GLB2 | CHRDL1 | FADD | OIP5 |
| SPANXA1 /// SPANXA2 /// SPANXB1 /// SPANXC | HBA1 /// HBA2 | GGH | LPIN1 |
| CLTB | TEAD4 | FAM107A | HSPD1 |
| SNX16 | XK | RORA | ACKR3 |
| CDC6 | PFN2 | SLC24A3 | ANP32E |
| BSPRY | LRP12 | NELL2 | SERPINB3 |
| PDZD2 | ATP10B | MFHAS1 | EPS8L2 |
| RHOBTB3 | LPCAT1 | APOBEC3B | TXNRD1 |
| STK39 | TGFBR3 | SLC1A4 | LPCAT1 |
| UPK3B | ACKR1 | S100A9 | IGLC1 |
| LRRFIP2 | DHRS9 | PKP3 | AHNAK |
| ASPN | COL4A1 | PRSS23 | SHCBP1 |
| LRP10 | AREG | LAMB1 | APOE |
| CENPN | HOXD11 | CAMK2N1 | LDB3 |
| FOXM1 | PDLIM2 | SELENBP1 | POPDC3 |
| PRSS23 | SPC25 | SORT1 | ALCAM |
| NRCAM | STIL | IFI16 | MT1M |
| ALDH9A1 | P2RY14 | ABHD5 | SFRP1 |
| NAGK | CLDN10 | PDLIM5 | RAI14 |
| ECHDC2 | YOD1 | DOCK9 | COL4A1 |
| DFNA5 | RAI14 | CDC25B | NFE2L3 |
| GOLM1 | WDHD1 | KIF20A | MUC1 |
| GEMIN2 | FN1 | ITPR3 | CBX3 |
| FBXO3 | TMEM45A | TIAM1 | KDELR3 |
| CYP4F3 | IGKC | MLF1 | NCAPG |
| PAX9 | MUC1 | KLK7 | FADD |
| CRYL1 | PXDN | LYPD3 | DBF4 |
| SLC38A6 | AGFG2 | CENPA /// SLC35F6 | HNMT |
| USP6NL /// USP6NL-IT1 | CSAG2 /// CSAG3 | COL4A1 | UPK3B |
| HNMT | CDC25B | IFI6 | MIR4680 /// PDCD4 |
| MAD2L1 | SLCO1B3 | C7 | PHACTR2 |
| IGLV1-44 | ITGA6 | TBL1XR1 | BAMBI |
| YOD1 | CCNB1 | ESPL1 | VAV3 |
| MCM10 | MTHFD2 | FRMD4B | TJP1 |
| SERPINB6 | EPS8L2 | TNFSF10 | HOMER3 |
| PAIP2B | ALDH3B2 | APMAP | S100A12 |
| HOXC10 | SHOX2 | RAB11A | GPR126 |
| RMND5B | ITM2A | PHACTR4 | ADAM12 |
| KIF20A | IDO1 | SH3GLB2 | PLA2G7 |
| NDC80 | PMM1 | PDZRN3 | SORT1 |
| PLAUR | LEPREL4 | OR7E14P | CNN3 |
| RAI14 | ALOX15B | DNMT1 | FOSB |
| NUAK1 | SLC39A14 | SECISBP2L | CYP2J2 |
| TIAM1 | ZBTB16 | SYNM | MMP2 |
| LPIN1 | HOXD10 | EFNA1 | CRYL1 |
| PMM1 | HPSE | PDZK1IP1 | ASPN |
| PRSS2 | MYO6 | VRK1 | TLR3 |
| GREM1 | CCNA2 | ARPC1B | GULP1 |
| ZBED2 | GMDS | ATP1A2 | COL7A1 |
| LAMB3 | IFI30 /// PIK3R2 | DUSP1 | VOPP1 |
| ARHGAP32 | CAST | SLC20A1 | AIM1 |
| IFI6 | CNN1 | TFF3 | LY96 |
| HSDL2 | CRYL1 | AGRN | HSDL2 |
| HOMER3 | DNMT3B | JAG2 | AIM1L |
| MYO5A | SLC38A6 | ANXA3 | PTH2R |
| RHOD | SORBS1 | IRS1 | LOX |
| CYAT1 /// IGLC1 /// IGLV1-44 | PALMD | C18orf25 | MAOA |
| FUT2 | ELOVL4 | MT1M | FADS1 /// MIR1908 |
| BGN | TMEM100 | SLC2A1 | MYO1B |
| SH3GLB1 | GINS2 | PLOD2 | RGS5 |
| MMP11 | NCAPG | NUP107 | PDLIM2 |
| UBE2C | STAT1 | KPNA2 | HOXD11 |
| DTL | ODC1 | SLC33A1 | IFI6 |
| PLD1 | LUM | THY1 | GOLM1 |
| CCL20 | CCNE2 | DHRS1 | ELOVL4 |
| SMYD3 | S100A14 | HSP90AA1 | RBP1 |
| MMD | IL36RN | UCHL1 | TMEM97 |
| CHST15 | KLK10 | MSH6 | ABHD5 |
| DSG3 | ZBED2 | ATP1B3 | FEN1 |
| ANXA1 | NDRG2 | VPS37B | SLC38A6 |
| EDN3 | GULP1 | ZNF426 | SPAG5 |
| COL4A5 | IGLV1-44 | IFI44L | RAD54B |
| TMEM97 | ARPC1B | RRAD | PXDN |
| BLM | IGKV1-17 | ACOX1 | DFNA5 |
| SLC16A1 | CYAT1 /// IGLC1 /// IGLV1-44 | FEN1 | TIMELESS |
| FADD | IGF2BP3 | FANCI | HJURP |
| CXCL1 | STK39 | FN1 | RNASE4 |
| SNX24 | OIP5 | IVL | CAST |
| ETHE1 | FST | TOPBP1 | PMEPA1 |
| AGA | IFI44 | AQP1 | KPNA2 |
| XK | ANP32E | LMNB1 | CCNA2 |
| CTSK | DUSP1 | CHRDL1 | SLC16A1 |
| PTGS1 | RMND5B | RANBP1 | IGKC |
| EPHX2 | ZFP36 | MYO10 | DKC1 |
| UGT1A1 | HBB | NBEAL2 | SPAG16 |
| CLIP1 | PHACTR2 | DPY19L4 | CNN1 |
| NPEPPS | CCL14 /// CCL15-CCL14 | HSPE1 | COX7A1 |
| DNMT3B | PLN | CYP2E1 | SMYD3 |
| MAOA | LY96 | RGS5 | GPNMB |
| GPNMB | THY1 | BUB1 | EPHX2 |
| FANCI | GCNT3 | MAGEA12 | DEPTOR |
| HMMR | S100A9 | BST2 | ECHDC2 |
| PLXNA1 | RNF141 | LRRC8D | PRSS2 |
| TPX2 | VOPP1 | CNN3 | ZBTB16 |
| DOCK9 | OLFML2B | NPEPPS | MCM7 |
| TF | FZD6 | RAD51AP1 | CENPE |
| FSCN1 | FAM189A2 | GMPS | PAICS |
| HERC5 | KIF18A | MXRA5 | FAM189A2 |
| ARNTL2 | MYO5A | ATP6V1C1 | EXO1 |
| INPP1 | MYO1B | CCT6A | KIF15 |
| ABCA8 | FAM63A | AURKB | KLHL7 |
| GRN | DSG2 | NNMT | IL36A |
| TECR | MAOB | ANP32E | PDZK1IP1 |
| USP46 | SLC2A1 | PTDSS1 | FZD6 |
| NID2 | EPHX2 | ANXA1 | EZH2 |
| LAMP3 | HJURP | PMM1 | MYOC |
| AGFG2 | KDELR3 | ALDH3B2 | GEMIN2 |
| NNMT | MMP2 | RACGAP1 | ACTL6A |
| GALNT6 | DOPEY2 | CPEB3 | TEAD4 |
| RIPK4 | PRSS3P2 | ACADM | YOD1 |
| CUL4B | MLF1 | LOC101927458 /// LPHN2 | ZWINT |
| GCLM | CBX3 | TMEM185B | ACTG2 |
| MYO6 | TJP3 | AZGP1 | ZFP36 |
| IL1R2 | CASQ2 | FUT2 | TDO2 |
| TMEM158 | BSPRY | TMX1 | SLCO1B3 |
| C3orf14 | FBN2 | ALDH9A1 | RAB11FIP1 |
| HIST1H2BC /// HIST1H2BE /// HIST1H2BF /// HIST1H2BG /// HIST1H2BI | E2F3 | PIM1 | NUAK1 |
| BAMBI | KCNS3 | KIF11 | FBXO3 |
| ACAA1 | MFHAS1 | RRAGD | CYAT1 /// IGLC1 /// IGLV1-44 |
| POF1B | RBP1 | ANXA11 | KIF18B |
| IGKC | ZWINT | ATR | POLE2 |
| PMEPA1 | CYP2C9 | MCM7 | MFHAS1 |
| KDELR3 | RERGL | CHSY1 | MLF1 |
| MGST2 | TNC | CSE1L | IGLV1-44 |
| DENND2D | PLAGL1 | E2F3 | PKP3 |
| TTK | COL7A1 | AGFG2 | RIOK3 |
| GINS1 | ALCAM | PELI1 | LRP8 |
| LOC101927458 /// LPHN2 | DDAH1 | PARP12 | SPANXA1 /// SPANXA2 /// SPANXB1 /// SPANXC |
| ASPM | CTTN | CXCL14 | PLXNA1 |
| SOCS1 | SOAT1 | SMTN | ATP6V1C1 |
| OSBPL10 | GOLM1 | COX7A1 | CCL20 |
| DUSP1 | USP6NL /// USP6NL-IT1 | PSMB9 | OGN |
| IFI44 | FRMD4B | ERBB3 | KIF11 |
| U2SURP | SPAG16 | NREP | DYNLT3 |
| VCAM1 | SLC39A6 | DYNLT3 | FYCO1 |
| CCNB2 | SHCBP1 | PTPRK | TMEM158 |
| AKR1B1 | ERBB3 | BOP1 /// MIR7112 | CDH19 |
| ITCH | MUC5B | SH3BP4 | MSANTD3-TMEFF1 /// TMEFF1 |
| SLC39A2 | TF | TNFAIP6 | TF |
| CDK14 | CENPE | LRP12 | GMNN |
| TM7SF2 | PLP1 | MMD | HPSE |
| TSPO | ELOVL6 | CRIP2 | SLC39A6 |
| DEPTOR | AIM1 | PRSS2 | GAS7 |
| CDC20 | TMSB15A /// TMSB15B | AMIGO2 | LMOD1 |
| CSTB | MSANTD3-TMEFF1 /// TMEFF1 | CLDN7 | DHRS9 |
| SEC14L1 | NUAK1 | IFI44 | SMAGP |
| CDC42EP3 | PLXNA1 | RBP1 | UBE2S |
| SLK | PRAME | SAMD9 | THBS1 |
| RASAL2 | POPDC3 | ZBTB16 | GSN |
| MAGEA12 | SOCS1 | CSTB | NCAPG2 |
| PLAGL1 | MAGEA1 | ADH7 | DSCC1 |
| LOX | GMNN | PTK7 | ETV5 |
| KIF2C | FADD | ATP1B1 | IFI44L |
| APOC1 | FGFBP1 | FXR1 | GALNT6 |
| GINS2 | MAGEA5 | DFNA5 | PMM1 |
| ARHGEF10L | SLC16A1 | PUS7 | CDK4 |
| CD59 | ACTG2 | MTERF3 | SLC20A1 |
| ATP6V1D | LMOD1 | LOX | COL4A2 |
| NMRK1 | SNAPC1 | SNAPC1 | CTSK |
| BUB1B | TIMELESS | HBA1 /// HBA2 | E2F3 |
| OLFM1 | PRSS21 | COBL | TPPP3 |
| GRB10 | ACOX1 | DES /// SUPT20H | SAMD9 |
| IFIT1 | DOCK9 | ACKR3 | NDRG2 |
| NHLH2 | DYNLT3 |  | LAMA3 |
| PLBD1 | CD207 |  | PRAME |
| HMGB3 | BLVRB |  | AKR1B1 |
| PRSS8 | LMNB1 |  | STK39 |
| BIRC5 | GGH |  | CST6 |
| NID1 | EXO1 |  | IL13RA1 |
| ERBB3 | NID2 |  | TMEM45A |
| CLDN7 | RHOD |  | OSBPL10 |
| CDC25B | FEN1 |  | FXYD1 |
| CENPF | NFE2L3 |  | CASQ2 |
| SLC6A8 | LRRFIP2 |  | SERPINB6 |
| FBN2 | IGKV1-37 |  | RMND5B |
| PPFIBP2 | PSMB9 |  | ARPC1B |
| LBH | GRB10 |  | LRRFIP2 |
| VPS37B | PRH1-PRR4 /// PRR4 |  | ACOX1 |
| CLCA2 | SPRR1B |  | SLC6A8 |
| GAS7 | ACTL6A |  | MANSC1 |
| CBX3 | ORC6 |  | ELOVL6 |
| ARAP2 /// LOC101928667 | HOMER3 |  | GLTP |
| AURKB | HLTF |  | LRP10 |
| HEBP2 | SPAG5 |  | ADORA2B |
| MAST4 | MAFF |  | KIF18A |
| HJURP | MANSC1 |  | CDC25B |
| SPAG1 | MAOA |  | RERGL |
| MCM7 | EZH2 |  | PHLDA1 |
| TSPAN6 | CLTB |  | HLTF |
| PRC1 | GCHFR |  | PLAGL1 |
| SHOX2 | VAV3 |  | EN1 |
| ASCC2 | KRT14 |  | FAM63A |
| CCDC6 | DBF4 |  | AOC3 |
| TMEM40 | FADS1 /// MIR1908 |  | SNAPC1 |
| RAB27B | COL14A1 |  | DMD |
| MYH11 | TIMP1 |  | GPM6B |
| STIL | GBP1 |  | CHST1 |
| CD207 | GPR110 |  | FRMD4B |
| YEATS2 | KPNA2 |  | TYMS |
| NSG1 | ECHDC2 |  | SH3GLB2 |
| EPHA1 | THBS1 |  | LEPREL4 |
| CYP2C9 | CNN3 |  | KCNAB1 |
| SLCO1B3 | DKC1 |  | LYPD3 |
| CPPED1 | WFDC1 |  | CHEK1 |
| MIR7110 /// PDIA5 | NNMT |  | PITX2 |
| HIGD1A | PTH2R |  | DHRS2 |
| IRS1 | FUT2 |  | ZDHHC13 |
| CKS2 | KIF18B |  | MKI67 |
| FSTL1 | RSAD2 |  | RAD54L |
| RAD54B | CCDC69 |  | SEL1L3 |
| RAB11A | ACOX3 |  | RNASEH2A |
| HERC6 | KIF11 |  | DHCR7 |
| DDX60 | RAB25 |  | RAD51 |
| HCAR3 | MCM4 |  | MCM4 |
| ALOX5 | CXCL5 |  | TSPAN8 |
| APOE | CCL18 |  | GCHFR |
| BICD1 | OGN |  | LMNB1 |
| ALOX15B | SOD2 |  | ADAMDEC1 |
| CXCL12 | LOC101927458 /// LPHN2 | | TNC |
| MIR6778 /// SHMT1 | KCNAB1 |  | NCBP2 |
| PIGN | CXCL6 |  | GRB10 |
| DEFB1 | MCM7 |  | GIPC2 |
| OIP5 | UGT1A1 |  | SLC12A8 |
| COBLL1 | ETHE1 |  | ITIH5 |
| FYCO1 | SAMD9 |  | SOAT1 |
| PDLIM5 | LRRC15 |  | FOXE1 |
| MYO1B | XCL1 /// XCL2 |  | SLC4A4 |
| ANXA11 | TPPP3 |  | NMU |
| AKIP1 /// NUAK2 | FXYD1 |  | ATP8A1 |
| FAM63A | NOX4 |  | RNF141 |
| SEL1L3 | GREM1 |  | ACOX3 |
| TRAM2 | SEL1L3 |  | RUVBL1 |
| CHST2 | BASP1 |  | BORA |
| IL36A | SYNM |  | FUT2 |
| LY6G6C | ZFAND5 |  | ALDH9A1 |
| DBF4 | CDC7 |  | ARHGAP8 |
| STX12 | CTSK |  | PNO1 |
| P4HA1 | FNDC4 |  | ENAH |
| ORC6 | LY6G6C |  | ZNF91 |
| GSN | LRP10 |  | TMSB15A /// TMSB15B |
| RUVBL1 | RASAL2 |  | CD207 |
| SLC20A1 | LAMA3 |  | STAT1 |
| ACTL6A | IL13RA1 |  | FBN2 |
| FBN1 | MAGEA2 |  | PCNA |
| SPTLC2 | TIAM1 |  | SHOX2 |
| TRIM29 | PDLIM5 |  | AREG |
| DSG2 | IGKC |  | BCAT1 |
| CRIP1 | LIMCH1 |  | FBXO5 |
| SLC25A32 | CDCA3 |  | IGKV1-17 |
| ELL2 | RNASE4 |  | NID2 |
| RASAL1 | IGKV1OR2-108 |  | AQP1 |
| GNA15 | MICB |  | SCNN1A |
| TRIM13 | ZSCAN18 |  | AGFG2 |
| ARPC1B | KLK6 |  | TNS1 |
| GTSE1 | TMEM40 |  | TBL1XR1 |
| OSR2 | ALDH9A1 |  | RBM47 |
| NBEAL2 | PDZK1IP1 |  | SEMA3C |
| DSCC1 | GPR126 |  | CBS |
| EZR | BCHE |  | SECISBP2L |
| TMEM57 | TK1 |  | MSH6 |
| ALS2CL | IL1A |  | MEIS1 |
| ESPL1 | KNTC1 |  | ERVMER34-1 |
| SPC25 | MINPP1 |  | HSPE1 |
| SPOCK1 | ALS2CL |  | MYO10 |
| HTRA1 | XCL1 |  | KNTC1 |
| FEN1 | NCAPG2 |  | CRIP1 |
| CDH3 | TP63 |  | DOPEY2 |
| NUSAP1 | KLK8 |  | CLDN1 |
| LAMB1 | ARG1 |  | ERBB3 |
| NAAA | SH3GLB2 |  | HERC5 |
| STC1 | TM7SF2 |  | ARTN |
| SYNGR1 | MMD |  | DNA2 |
| MFSD5 | SLC7A11 |  | PGF |
| POPDC3 | PANX1 |  | PUS7 |
| METTL7A | NRCAM |  | SNX16 |
| PCOLCE2 | IGHA1 |  | TIPIN |
| MAOB | TYMP |  | NCAPH |
| CBS | PHACTR4 |  | TK1 |
| NOD2 | CLDN17 |  | NUP107 |
| HSPD1 | ENAH |  | ABCC5 |
| RRM2 | CHEK1 |  | PRSS23 |
| CHRM3 | ARHGAP32 |  | ARHGAP32 |
| SFRP4 | ITPR2 |  | CHI3L1 |
| CXCL13 | ZDHHC13 |  | DOCK9 |
| SQRDL | IGLJ3 |  | TRIB2 |
| TRIB2 | GSN |  | IFI44 |
| PTH2R | PCNA |  | IFI30 /// PIK3R2 |
| ETV5 | PKP3 |  | NCF2 |
| PDCD6IP | FABP6 |  | SUCLG2 |
| ANXA8 /// ANXA8L1 | BORA |  | NAAA |
| DMXL1 | FZD2 |  | CDCA3 |
| SHANK2 | TWIST1 |  | FZD2 |
| PLOD3 | KIF15 |  | DMXL1 |
| GMNN | PDZRN3 |  | CDC45 |
| TPD52L1 | RAD51 |  | PGBD5 |
| NPAS2 | DFNA5 |  | C1orf112 |
| CCNA1 | VLDLR |  | MAGEA12 |
| SSFA2 | FBXO3 |  | BSPRY |
| SLC6A1 | TYMS |  | AGRN |
| TJP3 | DES |  | MAFF |
| DMXL2 | IGKV4-1 |  | STMN1 |
| TIMP3 | DDX60 |  | TMEM246 |
| ABCC5 | HERC5 |  | GLS |
| RUNX3 | CYP4F12 |  | LEPR /// LEPROT |
| UGT1A9 | MIR7110 /// PDIA5 |  | POF1B |
| NAPA | NMRK1 |  | RAB27B |
| GCNT3 | AMOTL2 |  | NUDT11 |
| STYK1 | IL27RA |  | AMOTL2 |
| AMIGO2 | RUNX3 |  | MMP7 |
| MMP9 | RBM47 |  | PID1 |
| ZFAND5 | AGRN |  | ARHGEF10L |
| PCP4 | HSPD1 |  | THY1 |
| ACADM | MAGEA4 |  | FAM60A |
| KRCC1 | SMC2 |  | NR3C2 |
| GLS | CEMIP |  | CXCL6 |
| GATM | POLE2 |  | LOC101927458 /// LPHN2 |
| ACTG2 | ITCH |  | YEATS2 |
| GALE | CBS |  | TMEM194A |
| CHFR | RTP4 |  | SLC27A6 |
| TMEM194A | USP46 |  | CYP2C9 |
| PINK1 | SERPINB6 |  | CXCL11 |
| PIM1 | C1QB |  | PDZRN3 |
| AHNAK2 | GAS7 |  | ACAA1 |
| FZD6 | SNX16 |  | OLFML2B |
| PRRG4 | ETV5 |  | ALOX15B |
| UBE2G1 | NCAPH |  | CLTB |
| GNAI3 | GPNMB |  | P4HA1 |
| VLDLR | RUNX1 |  | ALS2CL |
| CD55 | HSDL2 |  | WFDC1 |
| CARHSP1 | PTK7 |  | CUL4B |
| AMACR | GCLM |  | CDC7 |
| KIF18B | RAD54B |  | POLQ |
| ANP32E | TNS1 |  | MRGBP |
| DKC1 | NCBP2 |  | PPAP2C |
| VPS4B | PLD1 |  | USP6NL /// USP6NL-IT1 |
| GRAMD1C | CHI3L1 |  | U2SURP |
| KLK6 | POF1B |  | PAX9 |
| HOMER2 | RAD54L |  | PTPLAD1 |
| TNC | COX7A1 |  | MMD |
| KHDC1L | MYBL2 |  | ZWILCH |
| MREG | GABBR1 /// UBD |  | IGKV1OR2-108 |
| GAL | IFI35 |  | TJP3 |
| PLXDC2 | MYO10 |  | TM7SF2 |
| BLVRB | PARP12 |  | RUNX3 |
| IL1A | SLC25A32 |  | FNBP1 |
| RAPGEFL1 | HOXC6 |  | KRT8 |
| NDRG2 | CDK4 |  | SPOCK1 |
| NOL12 /// TRIOBP | METTL7A |  | HIGD1A |
| SLC39A6 | ATP6V1C1 |  | PBX1 |
| UNC13B | BCL2A1 |  | CST4 |
| PLTP | ARHGAP8 |  | ATP1B3 |
| ABCG1 | TMEM35 |  | SEC14L1 |
| DHCR7 | VCAM1 |  | NNMT |
| ZNF91 | SLC20A1 |  | RPP25 |
| SSBP2 | MEIS1 |  | PPFIBP2 |
| VRK1 | TLR2 |  | VRK1 |
| SMAP1 | NUP107 |  | TUSC3 |
| MINPP1 | MKI67 |  | KCNMA1 |
| IL36RN | PTGDS |  | TAF1A |
| MAPK13 | FBXO5 |  | MYBL2 |
| IGFBP7 | GALNT6 |  | ARL6IP5 |
| SMPDL3A | CTSC |  | MINPP1 |
| FAM216A | COL4A2 |  | MTERF3 |
| KRT15 | TRIM16 |  | EYA2 |
| SUCLG2 | DSC2 |  | KLF2 |
| PGD | PRSS23 |  | LGALS1 |
| TIPIN | SYNGR1 |  | PDPN |
| TUBB2A | TMEM246 |  | PAK2 |
| KLF6 | FUT8 |  | ENO2 |
| NETO2 | CHST2 |  | RUNX1 |
| SOD2 | KHDC1L |  | MCM6 |
| CBLB | RNASEH2A |  | CENPI |
| PITX2 | DSCC1 |  | ABCG1 |
| PPP1R7 | AQP1 |  | SEPP1 |
| LPAR6 | FYCO1 |  | SLC25A32 |
| PRKRIR | PIM1 |  | GPX2 |
| TWIST1 | BST2 |  | PEG3 |
| DPY19L4 | RASAL1 |  | LMNB2 |
| TPPP3 | LRP8 |  | BICD1 |
| MSH6 | PI3 |  | APMAP |
| PLAT | ITIH5 |  | VCAM1 |
| PDGFA | SLC12A8 |  | TIMP1 |
| TST | SLC6A1 |  | PDLIM5 |
| TMEM9B | U2SURP |  | SH3BGRL |
| TOLLIP | CLU |  | NUDT1 |
| S100A10 | ZNF107 |  | PTGS1 |
| LDHB | LY6D |  | CPPED1 |
| RAD54L | WARS |  | CENPM |
| TOM1 | PDPN |  | PRUNE2 |
| ARL6IP5 | NAGK |  | MRPS17 |
| ATP6V1C1 | RUVBL1 |  | KHDC1L |
| MOXD1 | P4HA1 |  | SELENBP1 |
| TMEM246 | ARHGEF10L |  | TMEM132A |
| CXCL11 | NR3C2 |  | GATM |
| SECISBP2L | APMAP |  | ILF2 |
| MYBL2 | TRIB2 |  | ANXA1 |
| LOXL1 | POLQ |  | MYO6 |
| PHACTR4 | AHNAK2 |  | FAT1 |
| TUSC3 | NPEPPS |  | HOXD10 |
| CHMP2B | CCNA1 |  | TIAM1 |
| SPAG5 | UGT1A9 |  | PDE2A |
| FTL | PPP1R1A |  | NUP155 |
| TWF1 | VPS37B |  | RFC3 |
| MAFF | MRGBP |  | COCH |
| SPINT1 | ESPL1 |  | ARHGAP6 |
| FZD2 | RFC3 |  | IL36RN |
| FAT1 | TMEM97 |  | SOCS1 |
| ADCY3 | CENPM |  | NMRK1 |
| TCF3 | ATP1B3 |  | FABP6 |
| ALAD | PAICS |  | FJX1 |
| WDHD1 | TMEM194A |  | MAGEA5 |
| TMEM254 | TOPBP1 |  | TCF3 |
| GNE | SLK |  | PPP1R1A |
| HDGFRP3 | KLF6 |  | PANX1 |
| OCLN | SLC27A6 |  | RHOD |
| CALU | CES1 /// LOC100653057 | | TOPBP1 |
| ARF6 | ARNTL2 |  | LMBRD1 |
| TMSB15A /// TMSB15B | SLC39A2 |  | XCL1 /// XCL2 |
| WWC1 | RAB27B |  | KIAA0232 |
| EPB41L4B | SNX24 |  | MLLT11 |
| HOXD11 | NAAA |  | BMP7 |
| LRBA | CD163 |  | SSBP2 |
| RALA | GLS |  | RFC5 |
| PPIC | APOL1 |  | DIEXF |
| KRT8 | RGS5 |  | COBLL1 |
| NFE2L3 | MIR6778 /// SHMT1 |  | SYNGR1 |
| TJP2 | IFITM1 /// IFITM2 |  | CDK14 |
| C7 | ST3GAL4 |  | HSPH1 |
| UPP1 | AKR1B1 |  | STX12 |
| RSAD2 | PLCD1 |  | RACGAP1 |
| JADE2 | IGLL3P |  | PSPH |
| KNTC1 | TXNRD1 |  | GREM2 |
| PELI1 | ADORA2B |  | PRSS3P2 |
| AGRN | MCM6 |  | VPS37B |
| MFAP5 | CDK14 |  | FAM149A |
| POLE2 | LRRC8D |  | AMIGO2 |
| RABGGTA | TLR3 |  | ADRB2 |
| BBIP1 | GZMB |  | PPAT |
| TXN | SPOCK1 |  | PTDSS1 |
| CDKN2A | TMEM132A |  | ZFAND5 |
| TIMP1 | MSH6 |  | PLOD3 |
| BHLHE40 | ENO2 |  | C6orf62 |
| RSRC1 | C6orf62 |  | DNMT1 |
| POLQ | VAT1 |  | CTSC |
| NUP107 | CDC45 |  | CEMIP |
| LMO2 | KIAA0232 |  | TBC1D31 |
| KIAA0232 | CSTA |  | S100A8 |
| SLC35C1 | LSG1 |  | SOD2 |
| TIMP2 | LGALS1 |  | CALU |
| ISLR | OSBPL10 |  | PINK1 |
| NUDT11 | TCEAL2 |  | RANBP1 |
| CDKN2AIP | STMN1 |  | LAMB1 |
| TBL1XR1 | FJX1 |  | DPY19L4 |
| AEBP1 | MTERF3 |  | PRKDC |
| FBXO5 | LAMB1 |  | RAB11A |
| PROCR | CD59 |  | SIX1 |
| PGF | DNMT1 |  | DSN1 |
| ASNS | EPHA1 |  | SMC2 |
| XCL1 /// XCL2 | ZWILCH |  | ACADM |
| BAG2 | ERVMER34-1 |  | PRSS21 |
| PLN | ATP6V1D |  | AZGP1 |
| LAMP2 | SUCLG2 |  | PGRMC2 |
| ELOVL1 /// MIR6734 | MAPT |  | MIR7110 /// PDIA5 |
| CENPE | AGA |  | SNX24 |
| CAPNS1 | FAM149A |  | C16orf95 |
| E2F3 | GEMIN2 |  | BLVRB |
| GLI2 | YEATS2 |  | IGKV1-37 |
| ITSN2 | AOC3 |  | TMEM35 |
| DUSP22 | STC1 |  | CXCL9 |
| THY1 | GPM6B |  | EFNA1 |
| CLOCK | HIGD1A |  | GCNT3 |
| SLC2A1 | CSTB |  | UNC13B |
| ARTN | PEG3 |  | DDX39A |
| HSPBAP1 | CHP1 |  | TWIST1 |
| MLLT11 | CUL4B |  | NAGK |
| ZFP36 | SLC39A8 |  | BOP1 /// MIR7112 |
| UGCG | SMYD3 |  | NPEPPS |
| XCL1 | APOD |  | RSAD2 |
| TNFAIP3 | HEBP2 |  | SHMT2 |
| IFITM1 /// IFITM2 | COBLL1 |  | CD59 |
| KRT6B | ATP6V0A4 |  | VLDLR |
| PPP1R13L | DHRS2 |  | HSPA2 |
| SLC7A1 | ARHGAP10 |  | SLC6A10P |
| RABGAP1L | FOXG1 |  | CHST2 |
| PDZD8 | MDK |  | EGFL6 |
| MEIS1 | SEMA3C |  | LRRC8D |
| CRAT | C1S |  | ACOX2 |
| LMBRD1 | MMP7 |  | RAP1A |
| SEMA3F | DHCR7 |  | IGF1R |
| AMOTL2 | KCNMB1 |  | TRAM2 |
| MTERF3 | ACAA1 |  | TMX4 |
| PARP12 | GIPC2 |  | JAG2 |
| CENPI | PDIA4 |  | MOXD1 |
| FAP | DSG3 |  | NOX4 |
| IFI35 | RPP25 |  | PROS1 |
| LOC101928830 /// LTA4H | JAG2 |  | BRCA1 |
| STK24 | SLC35C1 |  | MICB |
| CLCN3 | LHX2 |  | GNE |
| ANO10 | CALB2 |  | CACYBP |
| LARP6 | HSPE1 |  | ITSN2 |
| ATP1B3 | VRK1 |  | USP46 |
| MDC1 | ACOX2 |  | ITPR3 |
| FAM189A2 | PDE2A |  | FNDC4 |
| SLC26A2 | PLEKHG6 |  | ANXA11 |
| RNF39 | PLBD1 |  | C3orf14 |
| LSG1 | HSPBAP1 |  | ALAD |
| TEAD4 | TUSC3 |  | CNPPD1 |
| MYH10 | GFOD2 |  | LSG1 |
| CDK4 | UNC13B |  | SLC39A4 |
| PLEKHG6 | GNE |  | NONO |
| COCH | MCM5 |  | NOL12 /// TRIOBP |
| FARP1 | SECISBP2L |  | ADCY3 |
| KPNA2 | CDH19 |  | BASP1 |
| PBX1 | EPB41L4A |  | ZSCAN18 |
| VAT1 | DES /// SUPT20H |  | FAM64A |
| LAMC1 | PRUNE2 |  | CSE1L |
| KIAA0101 | DNAH17 |  | KLF9 |
| TIMELESS | ME1 |  | SPRY1 |
| EN1 | RND3 |  | PARP12 |
| MECOM | DEPTOR |  | POLR2H |
| DLX2 | TAP1 |  | HELLS |
| TGFBR3 | UBE2G1 |  | ETHE1 |
| SHCBP1 | FAT1 |  | MAGEA1 |
| RAD51 | ACADM |  | PLBD1 |
| PANX1 | TBC1D31 |  | CXorf57 |
| EML3 | KDELC1 |  | SLC6A1 |
| SOCS6 | AKIP1 /// NUAK2 |  | PTTG1 |
| RUFY3 | FCGR1B |  | STC2 |
| SEPP1 | SLC4A4 |  | PAK1 |
| CHEK1 | 10-Sep |  | LMO2 |
| GPRC5A | PLOD3 |  | CALM1 |
| FZD7 | HMGB3P1 |  | NUP62CL |
| FNDC4 | PELI1 |  | ARHGAP10 |
| HSD17B4 | DEPDC1 |  | SLK |
| LGALS3 | LYN |  | ASCC2 |
| PNO1 | RAB11A |  | NCAPD2 |
| MYBL1 | NUP155 |  | TRIM13 |
| DHRS2 | RMND5A |  | CHMP2B |
| PLIN3 | SLC13A4 |  | KDELC1 |
| SEMA4D | UPP1 |  | HMGN5 |
| FXR1 | PINK1 |  | CCDC6 |
| CCNE2 | RFC5 |  | MCM3 |
| DENND4C | SMTN |  | HDGFRP3 |
| NCF2 | MXRA5 |  | FAM129A |
| TRPS1 | CAPN5 |  | EPB41L4B |
| SH3BGRL | DDX39A |  | CCT6A |
| TOPBP1 | OASL |  | H2AFV |
| SOAT1 | STC2 |  | GPR110 |
| FUT8 | MGST2 |  | SLC3A2 |
| BMP7 | S100A8 |  | FARP1 |
| NCOA1 | SFRP1 |  | MIR3658 /// UCK2 |
| C1GALT1 | DPY19L4 |  | MUC5B |
| GALNT7 | TSPAN6 |  | S100B |
| TMEM45A | EPB41L4B |  | MAST4 |
| NT5C2 | SCG5 |  | CKAP2 |
| SLC25A36 | TIPIN |  | NDC1 |
| CBR3 | TRIM13 |  | RPA3 |
| ALMS1 | SNCAIP |  | DENND4C |
| STAT1 | ANXA11 |  | CYP24A1 |
| NREP | TCF3 |  | TMEM185B |
| NFATC1 | IGLC1 |  | CNTNAP2 |
| CTNND1 | NLRX1 |  | DPY19L1 |
| NCAPG | TSPAN8 |  | DEPDC1 |
| MSMO1 | CALM1 |  | TSC22D3 |
| S100A8 | KLF2 |  | BBIP1 |
| NCAPG2 | SEC14L1 |  | PRIM2 |
| TMED3 | AHR |  | HPRT1 |
| CLIC4 | NRG1 |  | CDT1 |
| SUSD4 | NCAPD2 |  | ALDH6A1 |
| DOK4 | EFNA1 |  | HEBP2 |
| LEPREL4 | PGF |  | LAMC1 |
| CA9 | FNBP1 |  | LSM5 |
| NPR3 | BCAT1 |  | PTGDS |
| ZCCHC6 | POU2AF1 |  | LPAR1 |
| RGS14 | ELL2 |  | XCL1 |
| KIF15 | NUP62CL |  | CDCA8 |
| PRSS3P2 | C1orf112 |  | RIF1 |
| CSGALNACT2 | HSPH1 |  | PIM1 |
| MTERF4 | LY6E |  | PER3 |
| OAS1 | HPRT1 |  | FAM216A |
| GPR110 | GATM |  | KLF6 |
| ADRB2 | ABCC5 |  | CLIP1 |
| CALML3 | IFIT1 |  | IL6ST |
| RAB2A | CCL20 |  | MCM5 |
| NDUFA4L2 | PP14571 |  | CDCA4 |
| TMEM132A | NBEAL2 |  | FERMT1 |
| CDS1 | TRAM2 |  | SSFA2 |
| CLEC3B /// EXOSC7 | PRKDC |  | NRG1 |
| H2AFY | GREM2 |  | MECOM |
| BCAP29 | CLIP1 |  | BRIX1 |
| BORA | ARTN |  | C1S |
| ELF3 | TST |  | GIMAP6 |
| EPHA2 | KLHL7 |  | CLU |
| STC2 | UBE2S |  | LY6G6C |
| MCM4 | KRT8 |  | PDIA4 |
| FABP6 | BRCA1 |  | TIPARP |
| RFC3 | RSRC1 |  | LY6E |
| MCM6 | IFITM1 |  | TMEM177 |
| CALM1 | PLOD1 |  | GAL |
| COL7A1 | PUS7 |  | SAP18 |
| MYO10 | TIPARP |  | PER2 |
| EMC3 | ZNF281 |  | HMGB3P1 |
| CCNA2 | FCER1G |  | KAL1 |
| CCNB1 | PTPLAD1 |  | ALDH2 |
| VAMP8 | SLAMF8 |  | HSPBAP1 |
| KLHL7 | ITPR3 |  | F2R |
| ENY2 | FAM129A |  | PDCD6IP |
| PTPLAD1 | FAM60A |  | ZNF107 |
| CNPPD1 | IL1R2 |  | SPAG1 |
| XYLT1 | HSP90AA1 |  | HOXC6 |
| IGF2 /// INS-IGF2 | EYA2 |  |  |
| NCBP2 | CST4 |  |  |
| STK3 | TAF1A |  |  |
| SCCPDH | CALU |  |  |
| MELK | EPYC |  |  |
| BLZF1 | PRKAR2B |  |  |
| SCP2 | TOR3A |  |  |
| NCAPH | RNF39 |  |  |
| TMEM38B | GALE |  |  |
| ITGB4 | PNO1 |  |  |
| DDX11 | PBX1 |  |  |
| ZNF232 | ALDH3A2 |  |  |
| CNTNAP2 | PID1 |  |  |
| FOSB | DNA2 |  |  |
| HIST1H2AC | IRS1 |  |  |
| OSTF1 | FOXL2 |  |  |
| FAS | TACC3 |  |  |
| TRIM16 | TPCN1 |  |  |
| HBA1 /// HBA2 | C3orf52 |  |  |
| SH3BGRL3 | PPAT |  |  |
| SNAPC1 | PDCD6IP |  |  |
| LRRC8D | CNPPD1 |  |  |
| RND3 | NFASC |  |  |
| ADAM23 | WHSC1 |  |  |
| NRN1 | KYNU |  |  |
| PER2 | CDKN2AIP |  |  |
| HSPE1 | LBH |  |  |
| LAMA3 | OAS2 |  |  |
| PEA15 | MAST4 |  |  |
| HMOX1 | WWC1 |  |  |
| F2R | SLC6A8 |  |  |
| TPCN1 | TGIF2 |  |  |
| TBC1D31 | ENY2 |  |  |
| ABCA5 | GLIPR1 |  |  |
| OXSR1 | NDC1 |  |  |
| SLC12A8 | LGALS7 /// LGALS7B |  |  |
| TACSTD2 |  |  |  |
| SNX3 |  |  |  |
| SH3BP4 |  |  |  |
| CAMSAP1 |  |  |  |
| ALDH3A2 |  |  |  |
| EIF2AK2 |  |  |  |
| PRKDC |  |  |  |
| PLLP |  |  |  |
| ATP8A1 |  |  |  |
| TMEM135 |  |  |  |
| IGKV1-17 |  |  |  |
| TK1 |  |  |  |
| HAT1 |  |  |  |
| STMN1 |  |  |  |
| APMAP |  |  |  |
| PCOLCE |  |  |  |
| MPC1 |  |  |  |
| CMAS |  |  |  |
| ELMO2 |  |  |  |
| ESRP2 /// MIR6773 |  |  |  |
| DNMT1 |  |  |  |
| ITGAV |  |  |  |
| MAGEA2 |  |  |  |
| CPT1A |  |  |  |
| ARF4 |  |  |  |
| NUP155 |  |  |  |
| 5-Mar |  |  |  |
| ACTR1A |  |  |  |
| LRP8 |  |  |  |
| JAK2 |  |  |  |
| RP2 |  |  |  |
| SCRN1 |  |  |  |
| TP63 |  |  |  |
| FLII |  |  |  |
| EXO1 |  |  |  |
| RER1 |  |  |  |
| MRGBP |  |  |  |
| DYNC1I1 |  |  |  |
| KDELC1 |  |  |  |
| CFD |  |  |  |
| C12orf29 |  |  |  |
| HMGCR |  |  |  |
| PTDSS1 |  |  |  |
| TPSB2 |  |  |  |
| 10-Sep |  |  |  |
| WNT5B |  |  |  |
| GOLGA2 |  |  |  |
| PLAG1 |  |  |  |
| DAAM1 |  |  |  |
| RFC5 |  |  |  |
| PPDPF |  |  |  |
| SLC6A10P |  |  |  |
| NLRX1 |  |  |  |
| ZFYVE21 |  |  |  |
| GJB5 |  |  |  |
| DNA2 |  |  |  |
| BHLHE41 |  |  |  |
| CDK19 |  |  |  |
| NEDD9 |  |  |  |
| MEGF9 |  |  |  |
| MRPS17 |  |  |  |
| TRAF5 |  |  |  |
| DACT1 |  |  |  |
| SAR1B |  |  |  |
| PGRMC2 |  |  |  |
| CDCA3 |  |  |  |
| STEAP1B |  |  |  |
| PAICS |  |  |  |
| C11orf24 |  |  |  |
| MCL1 |  |  |  |
| PCYOX1 |  |  |  |
| PSRC1 |  |  |  |
| SH3GL1 |  |  |  |
| ANKRD10 |  |  |  |
| CLDN4 |  |  |  |
| CEBPB |  |  |  |
| RMND5A |  |  |  |
| TAX1BP1 |  |  |  |
| SRPX2 |  |  |  |
| SRD5A1 |  |  |  |
| C1orf112 |  |  |  |
| UBE2S |  |  |  |
| CSE1L |  |  |  |
| TYMS |  |  |  |
| TMEM185B |  |  |  |
| CCDC69 |  |  |  |
| IFITM1 |  |  |  |
| TNFRSF10B |  |  |  |
| PLXNA2 |  |  |  |
| FADS3 |  |  |  |
| NAMPT |  |  |  |
| SPATS2 |  |  |  |
| CALCOCO2 |  |  |  |
| MAGEF1 |  |  |  |
| ACOT9 |  |  |  |
| VSIG10 |  |  |  |
| PLA2G7 |  |  |  |
| ACVR1 |  |  |  |
| MCM3 |  |  |  |
| ABR |  |  |  |
| TIPARP |  |  |  |
| DESI1 |  |  |  |
| ALDH1A3 |  |  |  |
| RAP1A |  |  |  |
| MTCL1 |  |  |  |
| PTK2 |  |  |  |
| USO1 |  |  |  |
| P2RY14 |  |  |  |
| CHP1 |  |  |  |
| CSAG2 /// CSAG3 |  |  |  |
| ORMDL2 |  |  |  |
| MAGEA1 |  |  |  |
| IGKV1-37 |  |  |  |
| ADAM12 |  |  |  |
| PCNA |  |  |  |
| BCL6 |  |  |  |
| CHODL |  |  |  |
| ITPR3 |  |  |  |
| DBNDD1 |  |  |  |
| EXOC1 |  |  |  |
| PTPRK |  |  |  |
| COL9A3 |  |  |  |
| LGR4 |  |  |  |
| TUBB2B |  |  |  |
| GGH |  |  |  |
| WNT4 |  |  |  |
| POLR2H |  |  |  |
| VPS13D |  |  |  |
| LRRC17 |  |  |  |
| ZWINT |  |  |  |
| CENPM |  |  |  |
| TPSAB1 |  |  |  |
| ERBB2 |  |  |  |
| EPN3 |  |  |  |
| SIX1 |  |  |  |
| GOLGA4 |  |  |  |
| PLOD2 |  |  |  |
| ME1 |  |  |  |
| CIT /// MIR1178 |  |  |  |
| ARL4D |  |  |  |
| HIST1H2AD |  |  |  |
| TICAM1 |  |  |  |
| IFI30 /// PIK3R2 |  |  |  |
| PLCD1 |  |  |  |
| ZDHHC3 |  |  |  |
| POU2AF1 |  |  |  |
| TUBB6 |  |  |  |
| JAG2 |  |  |  |
| CD99 |  |  |  |
| CD163 |  |  |  |
| GOLIM4 |  |  |  |
| PPT1 |  |  |  |
| SREK1IP1 |  |  |  |
| TM4SF1 |  |  |  |
| WASL |  |  |  |
| RAB5B |  |  |  |
| KIF18A |  |  |  |
| NASP |  |  |  |
| RHOA |  |  |  |
| MORC2 |  |  |  |
| GIPC2 |  |  |  |
| INHBB |  |  |  |
| PAK2 |  |  |  |
| RIF1 |  |  |  |
| RASGRP1 |  |  |  |
| CAPG |  |  |  |
| HSPH1 |  |  |  |
| CPNE3 |  |  |  |
| SH3YL1 |  |  |  |
| BST2 |  |  |  |
| DPY19L1 |  |  |  |
| TAF1A |  |  |  |
| TGS1 |  |  |  |
| PLEKHM1 |  |  |  |
| ALG9 |  |  |  |
| CBX6 |  |  |  |
| NOTCH2 |  |  |  |
| SAP18 |  |  |  |
| SORBS1 |  |  |  |
| FBXL5 |  |  |  |
| EZH2 |  |  |  |
| CASP4 |  |  |  |
| DDX11 |  |  |  |
| TPM4 |  |  |  |
| LY6E |  |  |  |
| GPR161 |  |  |  |
| CYP11A1 |  |  |  |
| MAGEA5 |  |  |  |
| TTI1 |  |  |  |
| RAB14 |  |  |  |
| GUCY1A3 |  |  |  |
| ACOT13 |  |  |  |
| KAL1 |  |  |  |
| PCSK5 |  |  |  |
| LYN |  |  |  |
| LPAR1 |  |  |  |
| PUS7 |  |  |  |
| AIM2 |  |  |  |
| ACADVL |  |  |  |
| NUDT1 |  |  |  |
| CXorf57 |  |  |  |
| ITGA5 |  |  |  |
| EPB41L4A |  |  |  |
| MIR6513 /// TMBIM1 |  |  |  |
| MSH2 |  |  |  |
| RPL29P7 /// RPL29P7 |  |  |  |
| FAM172A |  |  |  |
| TRA2B |  |  |  |
| PROS1 |  |  |  |
| FAM60A |  |  |  |
| ELK3 |  |  |  |
| MSRA |  |  |  |
| CACYBP |  |  |  |
| E2F6 |  |  |  |
| PLOD1 |  |  |  |
| GBAS |  |  |  |
| SCARB1 |  |  |  |
| UFL1 |  |  |  |
| ARPC2 |  |  |  |
| FHL1 |  |  |  |
| RARRES1 |  |  |  |
| CUX1 |  |  |  |
| DGKA |  |  |  |
| ACD |  |  |  |
| SLC12A7 |  |  |  |
| SAV1 |  |  |  |
| IGFBP4 |  |  |  |
| MKI67 |  |  |  |
| UBAP1 |  |  |  |
| TOR3A |  |  |  |
| RGS5 |  |  |  |
| CCSER2 |  |  |  |
| SERPINB9 |  |  |  |
| CYP1B1 |  |  |  |
| ASL |  |  |  |
| THAP10 |  |  |  |
| HBB |  |  |  |
| GALNT1 |  |  |  |
| ATP2C1 |  |  |  |
| TMX4 |  |  |  |
| HIST1H1C |  |  |  |
| CDK7 |  |  |  |
| SLC3A2 |  |  |  |
| TMEM159 |  |  |  |
| KLHL23 |  |  |  |
| EPHB4 |  |  |  |
| FOSL2 |  |  |  |
| KLF5 |  |  |  |
| LPHN3 |  |  |  |
| DIEXF |  |  |  |
| GINS4 |  |  |  |
| BAIAP2 |  |  |  |
| SLC27A6 |  |  |  |
| SDC2 |  |  |  |
| USP25 |  |  |  |
| STX7 |  |  |  |
| NCAPD2 |  |  |  |
| ITPKC |  |  |  |
| KDM6A |  |  |  |
| ADA |  |  |  |
| GMPS |  |  |  |
| IPO9 |  |  |  |
| MAF |  |  |  |
| MYO5C |  |  |  |
| TGM5 |  |  |  |
| BOP1 /// MIR7112 |  |  |  |
| CDC45 |  |  |  |
| MFSD1 |  |  |  |
| RAD21 |  |  |  |
| SLC13A4 |  |  |  |
| KLHL2 |  |  |  |
| DNAJB1 |  |  |  |
| TMPRSS4 |  |  |  |
| CHMP2A |  |  |  |
| HSPA2 |  |  |  |
| SLC33A1 |  |  |  |
| ZWILCH |  |  |  |
| TMEM80 |  |  |  |
| ALDH2 |  |  |  |
| ERVMER34-1 |  |  |  |
| TPSAB1 /// TPSB2 |  |  |  |
| PIK3R1 |  |  |  |
| POLR2K |  |  |  |
| RAD23B |  |  |  |
| CAPN1 |  |  |  |
| PIAS1 |  |  |  |
| AQP1 |  |  |  |
| RNASE4 |  |  |  |
| ARHGAP8 |  |  |  |
| DENND1B |  |  |  |
| SMPD2 |  |  |  |
| SSB |  |  |  |
| APOL1 |  |  |  |
| BRIX1 |  |  |  |
| FEZ1 |  |  |  |
| COL6A2 |  |  |  |
| POLA2 |  |  |  |
| AP3M2 |  |  |  |
| MOSPD1 |  |  |  |
| MAP3K9 |  |  |  |
| RNASEH2A |  |  |  |
| CERS2 |  |  |  |
| PPP1CB |  |  |  |
| CERS4 |  |  |  |
| MNAT1 |  |  |  |
| H2AFZ |  |  |  |
| CNGA1 |  |  |  |
| RC3H2 |  |  |  |
| PRNP |  |  |  |
| ZNF532 |  |  |  |
| IGF1R |  |  |  |
| ARHGAP10 |  |  |  |
| COX17 /// POPDC2 |  |  |  |
| IGKV1OR2-108 |  |  |  |
| CHKA |  |  |  |
| TSG101 |  |  |  |
| PRPS1 |  |  |  |
| CNN1 |  |  |  |
| WNK1 |  |  |  |
| FAM107A |  |  |  |
| ATP1A2 |  |  |  |
| PARP2 |  |  |  |
| SLC37A1 |  |  |  |
| P2RX5 |  |  |  |
| CAPN5 |  |  |  |
| PLK3 |  |  |  |
| DHCR24 |  |  |  |
| WDR26 |  |  |  |
| DDX39A |  |  |  |
| DRAM1 |  |  |  |
| FAM64A |  |  |  |
| TOB1 |  |  |  |
| TGIF2 |  |  |  |
| CFLAR |  |  |  |
| ILF3 |  |  |  |
| PSMB9 |  |  |  |
| DNM1 |  |  |  |
| RAB5A |  |  |  |
| IFIT3 |  |  |  |
| IMPAD1 |  |  |  |
| ATP2B1 |  |  |  |
| RPA3 |  |  |  |
| LSM5 |  |  |  |
| P2RY2 |  |  |  |
| MRE11A |  |  |  |
| ENSA |  |  |  |
| CSTA |  |  |  |
| PTP4A3 |  |  |  |
| LRP5 |  |  |  |
| ADAMDEC1 |  |  |  |
| HSP90AA1 |  |  |  |
| ST14 |  |  |  |
| ASAH1 |  |  |  |
| MAPKAPK3 |  |  |  |
| SLC19A1 |  |  |  |
| SP140L |  |  |  |
| DSN1 |  |  |  |
| FOXD1 |  |  |  |
| VWA5A |  |  |  |
| ZNF107 |  |  |  |
| CHST1 |  |  |  |
| MICAL2 |  |  |  |
|  |  |  |  |
